# Multi-Omics Investigations Revealed Underlying Molecular Mechanisms Associated With Tumor Stiffness and Identified Sunitinib as a Potential Therapy for Reducing Stiffness in Pituitary Adenomas

**DOI:** 10.3389/fcell.2022.820562

**Published:** 2022-03-15

**Authors:** Zihao Wang, Mengqi Chang, Yanruo Zhang, Gang Zhou, Penghao Liu, Jizhong Lou, Yuekun Wang, Yuan Zhang, Xiaopeng Guo, Yaning Wang, Xinjie Bao, Wei Lian, Yu Wang, Renzhi Wang, Wenbin Ma, Bing Xing, Jun Gao

**Affiliations:** ^1^ Department of Neurosurgery, Peking Union Medical College Hospital, Chinese Academy of Medical Sciences and Peking Union Medical College, Beijing, China; ^2^ State Key Laboratory of Complex Severe and Rare Diseases, Peking Union Medical College Hospital, Chinese Academy of Medical Science and Peking Union Medical College, Beijing, China; ^3^ Key Laboratory of RNA Biology, CAS Center for Excellence in Biomacromolecules, Institute of Biophysics, Chinese Academy of Sciences, Beijing, China; ^4^ University of Chinese Academy of Sciences, Beijing, China

**Keywords:** pituitary adenoma, stiffness-related gene, DNA methylation, m6A, sunitinib

## Abstract

**Purpose:** Pituitary adenomas (PAs) are the second most common intracranial neoplasms. Total surgical resection was extremely important for curing PAs, whereas tumor stiffness has gradually become the most critical factor affecting the resection rate in PAs. We aimed to investigate the molecular mechanisms of tumor stiffening and explore novel medications to reduce stiffness for improving surgical remission rates in PA patients.

**Methods:** RNA sequencing, whole-genome bisulfite sequencing, and whole exome sequencing were applied to identify transcriptomic, epigenomic, and genomic underpinnings among 11 soft and 11 stiff PA samples surgically resected from patients at Peking Union Medical College Hospital (PUMCH). GH3 cell line and xenograft PA model was used to demonstrate therapeutic effect of sunitinib, and atomic force microscopy (AFM) was used to detect the stiffness of tumors.

**Results:** Tumor microenvironment analyses and immunofluorescence staining indicated endothelial cells (ECs) and cancer-associated fibroblasts (CAFs) were more abundant in stiff PAs. Weighted gene coexpression network analysis identified the most critical stiffness-related gene (SRG) module, which was highly correlated with stiff phenotype, ECs and CAFs. Functional annotations suggested SRGs might regulate PA stiffness by regulating the development, differentiation, and apoptosis of ECs and CAFs and related molecular pathways. Aberrant DNA methylation and m6A RNA modifications were investigated to play crucial roles in regulating PA stiffness. Somatic mutation analysis revealed increased intratumoral heterogeneity and decreased response to immunotherapy in stiff tumors. Connectivity Map analysis of SRGs and pRRophetic algorithm based on drug sensitivity data of cancer cell lines finally determine sunitinib as a promising agent targeting stiff tumors. Sunitinib inhibited PA growth *in vitro* and *in vivo*, and also reduced tumor stiffness in xenograft PA models detected by AFM.

**Conclusion:** This is the first study investigating the underlying mechanisms contributing to the stiffening of PAs, and providing novel insights into medication therapy for stiff PAs.

## Introduction

Pituitary adenomas (PAs) are the second most common type of intracranial tumors, accounting for approximately 15% of primary central nervous system tumors (Ostrom et al., 2014). The mass effect of PA and secondary hypopituitarism and the multisystem complications caused by excessive secretion of hormones seriously reduce the quality of life and increase the mortality of PA patients (Molitch, 2017). During the past few decades, transsphenoidal surgery has been the first-line therapy for PAs, and only a few patients gain limited benefits from radiotherapy and medical treatments (Tabaee et al., 2009; Molitch, 2017; Almutairi et al., 2018). However, gross total resection can only be achieved in 66–78% of PA patients (Tabaee et al., 2009; Almutairi et al., 2018). Despite concurrent treatments, many patients still suffer repeated recurrence of tumors, with a 10-years recurrence rate as high as 7–12% (Reddy et al., 2011; Salomon et al., 2018). Hence, pursuing total resection of tumors during surgery and exploring new targeted drugs have become hopeful directions for reducing recurrence and curing PA patients. Due to the complicated anatomic structure in the sellar region and the limited operative field of view, it is very difficult for neurosurgeons to completely remove pituitary tumors with stiff texture, large size, and cavernous sinus invasion. In particular, tumor stiffness has become the most critical factor that affects the surgical resection rate despite the rapid progression of endoscopic surgery systems (Zhao et al., 2010; Sughrue et al., 2011). Soft tumors, even with larger size and cavernous sinus invasion, can be easily curetted through suctioning and usually have a better surgical outcome. Hence, achieving a better understanding of the underlying mechanisms of tumor stiffness and exploring novel medications to transform stiff tumors into soft ones are important for improving remission in PA patients.

Multiple studies have found that changes in tissue mechanical properties can both precede and drive disease treatment (Kai et al., 2016; Northcott et al., 2018), with tumor stiffness correlating with prognosis in several tumor types, including colorectal cancer, breast cancer and pancreatic ductal adenocarcinoma (Colpaert et al., 2001; Paszek et al., 2005; Kai et al., 2016; Laklai et al., 2016; Northcott et al., 2018; Anlaş and Nelson, 2020; Shen et al., 2020). At the tissue level, stiffness is governed by the cell cytoskeleton (Grady et al., 2016) and the extracellular matrix (ECM) (Humphrey et al., 2014). Fibrillar collagens are the most abundant ECM scaffolding proteins and contribute significantly to tissue stiffness (Mouw et al., 2014). Aberrant ECM remodeling with collagen I (COL-I) enrichment have been identified as major causes of tissue stiffening during cancer progression (Levental et al., 2009; Pickup et al., 2014). As the major source of ECM, CAFs further modify the tumor mechanical environment by expressing lysyl oxidase (LOX), an amine oxidase that initiates the process of covalent intramolecular and intermolecular crosslinking of collagen (Kagan and Li, 2003; Levental et al., 2009). In experimental models, inhibiting matrix stiffening via LOX inhibition ameliorates tumor growth and improves therapy (Levental et al., 2009). Thus, CAFs are regarded as a promising therapeutic target for limiting cancer progression (Pickup et al., 2014). Despite these findings, the molecular mechanisms that regulate the mechanical properties and contribute to stiffening in PAs still await elucidation.

Additionally, PA stiffness can be predicted through magnetic resonance imaging (MRI). Several studies testing the ability of MRI strategies utilizing machine learning algorithms to predict the tissue consistency of PAs have been performed (Hughes et al., 2016; Fan et al., 2019; Yao et al., 2020). Such strategies can help to determine individualized therapeutic schemes for PA patients. For those patients predicted to have stiff tumors, preferential use of medications that reduce tumor stiffness preoperatively might significantly improve the surgical resection rate.

In this study, by performing comprehensive multi-omics analyses of transcriptomic, genomic, DNA methylation, and m6A RNA methylation data from soft and stiff tumors, we aimed to explore the specific alterations associated with the unique biology of stiff tumors and the underlying mechanisms contributing to the stiffening of PAs. Furthermore, the responses to targeted therapy and immunotherapy were also explored to demonstrate the potential therapeutic value of treating stiff PA tumors. Sunitinib, the most critical predicted drug, was applied to treat PAs *in vitro* and *in vivo* in order to explore whether sunitinib could inhibit tumor growth and reduce stiffness.

## Materials and Methods

### Human Samples

We prospectively enrolled 30 patients with nonfunctioning PAs who underwent transsphenoidal adenectomy at Peking Union Medical College Hospital (PUMCH) between April 2018 and June 2018. All patients received pituitary endocrinological and neuroradiological evaluations before and after surgery and were diagnosed with nonfunctioning PAs confirmed by postoperative histopathology. The inclusion criteria were as follows: 1) intact and available perioperative endocrine examinations and sellar MRI; and 2) no history of pituitary surgery, radiotherapy or medical treatment (e.g., bromocriptine or cabergoline) preoperatively. During the surgical procedure, the consistency of the pituitary tumor was classified as stiff or soft by at least two of four experienced neurosurgeons together (Bahuleyan et al., 2006). In contrast to that of other solid tumors, the stiffness of PAs can be easily distinguished in most instances. Soft pituitary tumors are pasty, loose, and amenable to being suctioned out piecemeal with an aspirator, whereas stiff pituitary tumors grow to be almost firm spherical ellipsoids and cannot be removed easily with suction despite vigorous movement of the suction tips (Bahuleyan et al., 2006). Postoperatively, fresh tumor specimens were immediately frozen in liquid nitrogen and then stored at −80°C. After rigorous screening according to the inclusion criteria, 22 samples, including 11 soft and 11 stiff pituitary tumors, were used for integrated transcriptomic, genomic, and epigenomic profiling analyses. This study was approved by the Institutional Ethics Committee and Institutional Review Board of PUMCH (No. S-K431), and informed consent was obtained from all participants for the publication of any potentially identifiable images or data included in the study.

### DNA and RNA Extraction

Pituitary tumor samples (10–50 mg) were powdered under liquid nitrogen. DNA was extracted and purified following proteinase K digestion using a TIANamp Genomic DNA Kit (TIANGEN Biotech, Catalog No. DP304-03). DNA concentrations were determined using a Qubit DNA HS Assay Kit (Thermo Fisher, Catalog No. Q32854). The DNA quality was assessed with Agilent 2,200 TapeStation Genomic DNA Analysis (Agilent Technologies).

Total RNA was extracted using TRIzol reagent (Thermo Fisher, Catalog No. 15596018). The RNA quality was checked by Agilent 2,200 TapeStation RNA Analysis (Agilent Technologies) to determine the RNA integrity number (RIN). The RNA was considered acceptable for cDNA library construction when the RIN was >7.0.

### RNA Sequencing

cDNA libraries were constructed for each RNA sample using the TruSeq Stranded mRNA Library Prep Kit (Illumina, Inc.) according to the manufacturer’s protocols. The quality of the cDNA libraries was assessed with the Agilent 2,200 system, and the libraries were sequenced with the HiSeq X Ten system (Illumina, Inc.) with a 150 bp paired-end run. Raw reads were filtered with FAST-QC. Before read mapping, clean reads were obtained from the raw reads by removing the adaptor sequences and low-quality reads. The clean reads were then mapped to the human genome hg19 sequence (GRCh37) using HISAT2 (Kim et al., 2015). HTseq was used to generate gene counts, and the RPKM method was used to determine gene expression (Anders et al., 2015).

### Whole-Genome Bisulfite Sequencing

Genomic DNA was bisulfite converted with the EZ DNA Methylation-Gold Kit (Zymo Research) and then processed with the TruSeq DNA Methylation Kit (Illumina, Inc.) according to the manufacturer’s instructions for WGBS library construction. The tagged WGBS libraries were used for 150 bp paired-end sequencing in a single lane of the HiSeq X Ten system (Illumina, Inc.) with 10-15% phi-X for base balance. Before read mapping, clean reads were obtained from the raw reads by removing the adapters and sequences at the 5′ and 3′ ends with methylation bias by using Trim Galore. The bisulfite mapping of methylation sites, including alignment to human genome hg19 (GRCh37), removal of duplicates, and extraction of clean reads to a CpG count matrix, was performed by utilizing Bismark and then indexed by Bowtie2 (Krueger and Andrews, 2011; Langmead and Salzberg, 2012).

### Exome Sequencing and Variant Calling

To generate standard exome capture libraries, we used the Agilent SureSelectXT Reagent Kit (Agilent, Catalog No. G9611B) protocol for the Illumina HiSeq Paired-end Sequencing Library, SureSelectXT Human All Exon v6 system (Agilent, Catalog No. 5190-8864), followed by sequencing of libraries using paired-end mode (2 × 75 bp) on the HiSeq X Ten (Illumina, Inc.). Raw reads were filtered and evaluated with FAST-QC. Before read mapping, clean reads were obtained from the raw reads by removing the adaptor sequences, reads with >5% ambiguous bases (noted as Ns) and low-quality reads containing more than 20% bases with qualities of <20. The clean reads were then aligned to the human genome hg19 sequence (GRCh37) using BWA-MEM with the default settings (Houtgast et al., 2018). Variant calling was performed by using the GATK3 (version 3.6) standard pipeline with default parameters based on the mapping bam file, which was sorted, indexed and deduplicated with SAMtools and recalibrated with GATK tools (McKenna et al., 2010; Li, 2011). Mutation sites were annotated by the VEP analysis pipeline and filtered by the following criteria: allele frequency <0.01 in common frequency databases, such as gnomAD; IMAPCT over moderate; mutation frequency >0.1; sequencing depth >10; intolerant or damaging impact on protein structure and function predicted by the SIFT and PolyPhen-2 databases (McLaren et al., 2016; Amadori et al., 2020).

### Analysis of the Tumor Microenvironment and Immunogenomic Patterns of PAs

To clarify the cellular components of the PA TME, the xCell algorithm was employed to accurately quantify the abundances of 64 cell types within the admixtures of tumor samples by using the xCell package in R (Aran et al., 2017). The enrichment levels of 7 subgroups of epithelial cells, nine subgroups of hematopoietic progenitors, 21 subgroups of lymphoid cells, 13 subgroups of myeloid cells, and 14 subgroups of stromal cells were estimated via single-sample gene set enrichment analysis (ssGSEA) based on the gene expression profiles of PAs (Hänzelmann et al., 2013). The Estimation of Stromal and Immune Cells in Malignant Tumors using Expression Data (ESTIMATE) algorithm was utilized to assess factors of the overall TME, including the abundances of intratumoral stromal and immune cells and tumor purity, based on the transcriptomic profiles of PA samples (Yoshihara et al., 2013). The immune and stromal scores were used to represent the abundances of immune and stromal cells, the ESTIMATE score were used to represent general nontumoral components, and tumor purity was used to reflect general tumoral proportions. CIBERSORT, a deconvolution algorithm based on linear support vector regression, was also utilized to quantify the abundances of 22 subtypes of tumor-infiltrating immune cells (TIICs) in PAs (Newman et al., 2015). Furthermore, 31 immune signatures (gene sets) introduced by He et al. (2018) were utilized to reflect the overall immune activity of solid tumors in terms of the types, related immune functions and molecular pathways of TIICs, and these factors were quantified by the ssGSEA algorithm (Supplementary Table S1). Then, based on the enrichment scores of those immunogenomic signatures, unsupervised hierarchical clustering was performed to categorize PA patients into different clusters according to immune subtype. The high immunity group included “immune hot” tumors, which had the highest enrichment scores; the low immunity group included “immune cold” tumors, which had the lowest enrichment scores; and the medium immunity group included “immune altered” tumors, which had the potential to transform into hot or cold tumors (Wang et al., 2021). Additionally, to elucidate the functional heterogeneity of cancer cells at single-cell resolution, the gene signature profiles of 14 functional states of cancer cells obtained from CancerSEA were evaluated by ssGSEA using transcriptomics data from PA samples (Supplementary Table S1) (Yuan et al., 2019).

### Immunofluorescence Analysis

Immunofluorescence (IF) staining was performed on formalin-fixed, paraffin-embedded (FFPE) sections of tumor tissues. Briefly, FFPE tissues were cut into 4 μm sections, followed by deparaffinization and rehydration using xylene and ethanol. Next, the slides were incubated in EDTA antigen retrieval buffer at subboiling temperature and then in blocking solution (BSA; G5001, Servicebio) for 30 min at room temperature. The slides were incubated overnight with primary antibodies, including anti-CD31 (ab28364, Abcam), anti-von Willebrand factor (VWF) (bs-10048R, Bioss), anti-smooth muscle actin-α (αSMA) (A2547, Merck), and anti-S100A4 (bs-3759R, Bioss) antibodies, followed by incubation with fluorochrome-conjugated secondary antibodies for 50 min at room temperature. DAPI (G1012, Servicebio) was used to stain cell nuclei. Pictures were taken with an Ortho fluorescence microscope (ECLIPSE C1, NIKON). All staining was quantified using NIH ImageJ 1.51s analysis software with the same threshold for each stain.

### Gene Set Variation Analysis

GSVA was utilized to evaluate the 50 most significantly enriched hallmark pathways obtained from the Molecular Signatures database (MSigDB) (Subramanian et al., 2005) in soft and stiff tumors by using the GSVA package in R (Hänzelmann et al., 2013). Differential analysis of the enrichment scores of molecular pathways between two groups was performed with the limma package in R (Smyth et al., 2005). The hallmark pathways with |t value| > 4, indicating a false discovery rate (FDR) < 0.05, were considered the most differentially enriched molecular pathways between the two groups (Lambrechts et al., 2018).

### Weighted Gene Coexpression Network Analysis

The differentially expressed genes (DEGs) between soft and stiff tumors were identified by using the edgeR package in R based on the raw count data (Robinson et al., 2010). The criteria for selecting DEGs were Benjamini–Hochberg corrected FDR <0.05 and |fold change (FC)| > 2. Next, the coexpression network for the DEGs was constructed by the WGCNA package in R based on the RPKM data (Langfelder and Horvath, 2008). First, sample clustering was performed to detect outliers. The pickSoftThreshold method was used to select an appropriate soft threshold (power) to achieve a scale-free topology fit index >0.85 and maintain optimal mean connectivity. Afterwards, the adjacency matrix was transformed into a topological overlap matrix (TOM) to define gene coexpression similarity, and gene hierarchical clustering for TOM-based dissimilarity was performed to obtain the hierarchical clustering dendrogram. The Dynamic Tree Cut package was used to identify the modules with a minimum gene size of 50, and then the similarity cut-off was set to 0.75 to merge the modules after calculating the dissimilarity of module eigengenes (MEs), representing the overall expression profiles of each module. The adjacency of the MEs of all modules was determined by Pearson correlation analysis.

### Identification of Clinically Significant Modules

Pearson correlation analyses between MEs and clinical traits were performed to determine the key gene signatures associated with the stiffness of tumors. Furthermore, gene significance (GS) was calculated as the absolute value of the correlation between each gene within MEs and each trait, and module membership (MM) was calculated as the correlation of the gene expression profile and each ME. A high correlation between GS and MM suggested a highly significant association between the modules and clinical traits (Zhang and Horvath, 2005).

### Construction of the Protein-Protein Interaction Network and Functional Enrichment Analysis of Key MEs

The genes within the key module associated with stiffness were mapped with the STRING database (http://string-db.org) to evaluate their functional associations, and a combined score >0.4 was defined as significant (Mering et al., 2003). The PPI network, representing the topology of the interactions between SRGs, was constructed and visualized by Gephi software. To further explore the biological properties and molecular mechanisms of the SRGs, gene ontology (GO) enrichment analyses were performed with the ClueGO plug-in of Cytoscape (Bindea et al., 2009). An FDR <0.05 was considered significant.

### Identification of Differentially Methylated Regions and DMR-Associated Genes

DMRs between soft and stiff tumors were identified by using the DSS package in R (Park and Wu, 2016). First, the DMLtest function was used to perform statistical tests for differentially methylated loci (DML), including estimating mean methylation levels for all CpG sites and dispersions at each CpG site and performing the Wald test. Based on the DML results, the callDMR function was applied to identify DMRs consisting of at least four statistically significant CpG sites. A |delta| > 0.1 and *p* < 0.01 were considered the thresholds for determining DMLs and DMRs. Genes overlapping with DMRs in the whole genome were considered DMR-associated genes.

### Evaluation of m6A Modification

To explore the potential roles of RNA modifications in regulating the stiffness of PAs, three types of m6A regulators, including 2 demethylases (erasers), 7 methyltransferases (writers), and 11 RNA binding proteins (readers), were compared between soft and stiff tumors (Li et al., 2019). The post-methylation regulation of stiffness-related mRNAs was evaluated by Pearson analysis of the correlation between the expression of readers and SRGs. The overall effects of m6A in regulating the stiffness of PAs were comprehensively assessed as reported in the literature (He et al., 2019).

### Somatic Mutation Analysis

The different genomic variations between soft and stiff tumors were explored by somatic mutation analysis. The mutation types and frequencies of the top mutated genes were visualized by a waterfall plot using the maftools R package (Mayakonda et al., 2018). The differentially mutated genes (DMGs) between soft and stiff tumors were detected by the mafComapre function, which used Fisher’s test to compare all genes between the two groups. An FDR <0.05 was considered statistically significant. As reported by previous studies, mutation of transcription factors (TFs) plays a critical role in the development and progression of multiple cancers. The differentially mutated TFs were visualized by a lollipop plot. The target genes of TFs were obtained from the Gene Transcription Regulation database (GTRD, http://gtrd.biouml.org/) based on the ChIP-seq data. Tumor mutation burden (TMB) was defined as the total number of nonsynonymous mutations in the coding region per megabase (Budczies et al., 2018). Tumor heterogeneity was inferred by clustering variant allele frequencies (VAFs) using the inferHeterogeneity function, which clustered variants to infer clonality. According to the VAF clustering, clones were defined as either mutations that occurred in most cancer cells or that only existed in a small number of cells (subclones). The mutant-allele tumor heterogeneity (MATH) score is a novel quantitative measure of intratumoral genetic heterogeneity that calculates the width of the VAF distribution (Mroz et al., 2015). Tumors with high heterogeneity and a high subclonal fraction tend to experience immune evasion and resist immunotherapy (Guan and Shastri, 2018).

### Prediction of Targeted Drugs for PAs and Therapeutic Response

The Connectivity Map (CMap) database (https://clue.io/) was employed to explore potential compounds targeting the molecular pathways and genes associated with the stiffness of PAs (Subramanian et al., 2017). The SRGs of stiff tumors were considered potential targets of compounds used to query the CMap database. The enrichment scores of compounds were calculated, and compounds with enrichment scores < -95 and *p* < 0.05 were considered potential therapeutic drugs for stiff PAs. The most enriched mode of action (MoA) and corresponding drugs were selected for further analysis. Drug sensitivity data of cancer cell lines obtained from the Cancer Cell Line Encyclopedia (CCLE) project were obtained from the Genomics of Drug Sensitivity in Cancer database (GDSC, https://www.cancerrxgene.org/) (Geeleher et al., 2014a). These data provide the half maximal inhibitory concentration (IC50) as the measure of drug sensitivity, and lower IC50 values indicate increased sensitivity to compounds. After integrating the gene expression profiles of cell lines (as the training set) and PA samples (as the test set), the IC50 values of drugs in PA patients were estimated by ridge regression analysis via the pRRophetic R package, and the prediction accuracy was assessed by 10-fold cross-validation (Geeleher et al., 2014b).

### Prediction of the Response of PAs to Immunotherapy

The Tumor Immune Dysfunction and Exclusion (TIDE, http://tide.dfci.harvard.edu/) algorithm quantifies T cell dysfunction signatures by testing how the expression of each gene in solid tumors affects the infiltration of cytotoxic T lymphocytes (CTLs) to influence the immunotherapy response (Jiang et al., 2018). TIDE scores, which are suggestive of the clinical response to immunotherapy, were calculated based on the gene expression profiles of PA samples. A TIDE score <0 was considered to indicate sensitivity to immunotherapy, and >0 was considered to indicate resistance to immunotherapy. In addition, an unsupervised subclass mapping (https://cloud.genepattern.org/gp/) method was utilized to predict the clinical response to anti-PD1 and anti-CTLA4 therapy in soft and stiff tumors (Hoshida et al., 2007). An FDR <0.05 was considered to indicate a significant response to immune checkpoint inhibitors (ICIs).

### Cell Culture

The GH3 cell line were purchased from National Infrastructure of Cell Line Resource (Beijing, China) and cultured in DMEM/F12 medium supplemented with 15% horse serum, 2.5% fetal bovine serum, and an antibiotic-antimycotic solution. Cell cultures were maintained in an incubator at 37°C and 5% CO_2_.

### Cell Viability Assay

For Cell Counting Kit-8 (CCK-8) cell viability assays, cells were seeded in 96-well plates at a density of 5,000 cells per well and then incubated with dimethyl sulfoxide (DMSO; control) or a series of 2-fold-diluted concentrations of sunitinib (cat. no. S7781, Selleck) for 2 days. Six parallel wells were used for each concentration of the drugs. After incubation, CCK-8 assays (cat. no. CCK-8, Dojindo) were performed to measure cell viability according to the manufacturer’s instructions.

### Xenograft Experiment

A total of 12 Wistar Furth rats were used for generation of the GH3 xenograft model. Four-week-old female rats were subcutaneously injected with GH3 cells (5 × 105) into the left lumbar area. Tumor size was measured every 3 days, and tumor volume was calculated as width2 × length × 0.5. Twelve rats were randomly divided into 2 groups, including drug treatment group and control group. sunitinib was intragastrically administered at a dose of 40 mg/kg once daily when the tumor volumes approached approximately 100 mm^3^. All rats were sacrificed upon completion of the 12-days experiment, and the tumors were excised, weighed, and maintained in PBS at 4°C for stiffness analysis. All animal experiments and euthanasia were approved by Animal Care and Use Committee of PUMCH.

### Atomic Force Microscopy-Based Young’s Modulus Measurement of Tumor Samples

Resected xenograft PA samples were cut to proper size by a scalpel and immobilized on 6 cm-cell culture dishes (Thermofisher, cat. no. 15462) with two slices of 1mm× 5 cm-parafilm (Bemis, PM-996). Then the tissues were immersed in 1× PBS, and applied to the AFM force-measuring setting. A homemade AFM from Institute of Biophysics, Chinese Academy of Sciences was used in this study. Using constant force mode, more than 2000 force-to-distance traces (force curves) were recorded and more than 300 traces were selected for each group. The slope K (pN/nm) of every trace was calculated and then transformed to Young’s modulus (kilopascal, kPa) by using JPK Data Processing software. The final Young’s modulus of each tumor was calculated taking into account all traces recorded for a single tissue sample. The tumor stiffness between sunitinib treatment and control group were compared by mean Young’s modulus of each group, which was calculated by the Gaussian fitting *via* the amplitude version of Gaussian peak function (GaussAmp) in Origin software.

### Statistical Analyses

Independent Student’s t test was utilized for continuous variables and the χ2 test was utilized for categorical variables when making comparisons between two groups. The Mann-Whitney *U* test was used to compare categorical variables and nonnormally distributed variables between two groups. The Kruskal–Wallis test was used to compare multiple groups. Correlation analysis was performed by the Pearson correlation test, and a *p* value <0.05 and |correlation coefficient| > 0.3 were considered to indicate significant correlation. The statistical analyses in this study were performed with R 3.6.1 software. A two-tailed *p* value <0.05 was considered to indicate statistical significance.

## Results

### Clinicopathological Features of Soft and Stiff PAs

The demographics and clinicopathological features of 22 PA patients are summarized in Table 1. In general, almost all the clinical variables did not differ significantly between soft and stiff tumors. Gross total resections were achieved in all soft PAs (100%), whereas 18.2% of stiff tumors were only subtotally resected via transsphenoidal surgery due to firm texture. After a long-term follow-up (2.35 ± 0.06 years), recurrence occurred in three patients (all from the stiff tumor group), with an average recurrence time of 1.30 years. The recurrence in two of these patients was due to the active growth of residual tumors.

**TABLE 1:** Demographics and clinicopathological features of 22 patients diagnosed with pituitary adenomas.

Variables	Soft tumor (n = 11)	Stiff tumor (n = 11)	*p* Value
Age (years)	46.5 ± 14.7	47.7 ± 13.7	0.847
Gender (M/F)	6/5	8/3	0.659
Disease course (years)	0.67 (0.17, 2)	0.67 (0.25, 3)	0.597
No. of chief complaints	2 (1, 3)	2 (0, 2)	0.407
Endocrine examinations			
APD (N/Y)	7/4	6/5	1.0
Hyperprolactinemia (N/Y)	6/5	7/4	1.0
Pituitary MRI			
Max diameter (cm)	2.9 ± 0.6	3.4 ± 0.8	0.123
Tumor volume (ml)	7.6 ± 3.7	11.8 ± 8.9	0.158
Tumor size (macro/giant)	7/4	4/7	0.395
Apoplexy (N/Y)	7/4	10/1	0.311
Knosp classification (Grade II/III/IV)	5/5/1	5/3/3	0.472
Hardy classification (Grade II/III/IV)	6/4/1	8/2/1	0.621
Hardy classification (Type A/B/C-E)	8/3/0	5/6/0	0.387
Invasiveness (invasive/noninvasive)	6/5	6/5	1.0
Resection extent (GTR/STR)	11/0	9/2	0.476
Histopathology			
Pathological subtype (SSA/SGA)	9/2	6/5	0.361
Ki-67 index (%)	2 (1, 3)	1 (1, 2)	0.219
No. of recurrence (%)	0 (0)	3 (27.3)	0.214

AbbreviationsM/F, male/female; No., number; APD, anterior pituitary deficiency; N/Y, no/yes; GTR, gross total resection; STR, subtotal resection; SSA, silent somatotroph adenoma; SGA, silent gonadotroph adenoma.

Tumor volume = sagittal×coronal×axial diameters×π/6 (ml). Tumor size was classified as microadenoma (<1 cm), macroadenoma (1–4 cm) or giant pituitary adenoma (>4 cm). Invasiveness was determined by the following criterion: Knosp classification Grade III-IV, and Hardy classification Grade III-IV, and/or Type D-E according to the coronal view of sellar MRI. Disease course, number of chief complaints, and Ki-67 index are expressed as the median (interquartile range).

### ECs and CAFs Were More Abundant in Stiff PAs

**FIGURE 1:**
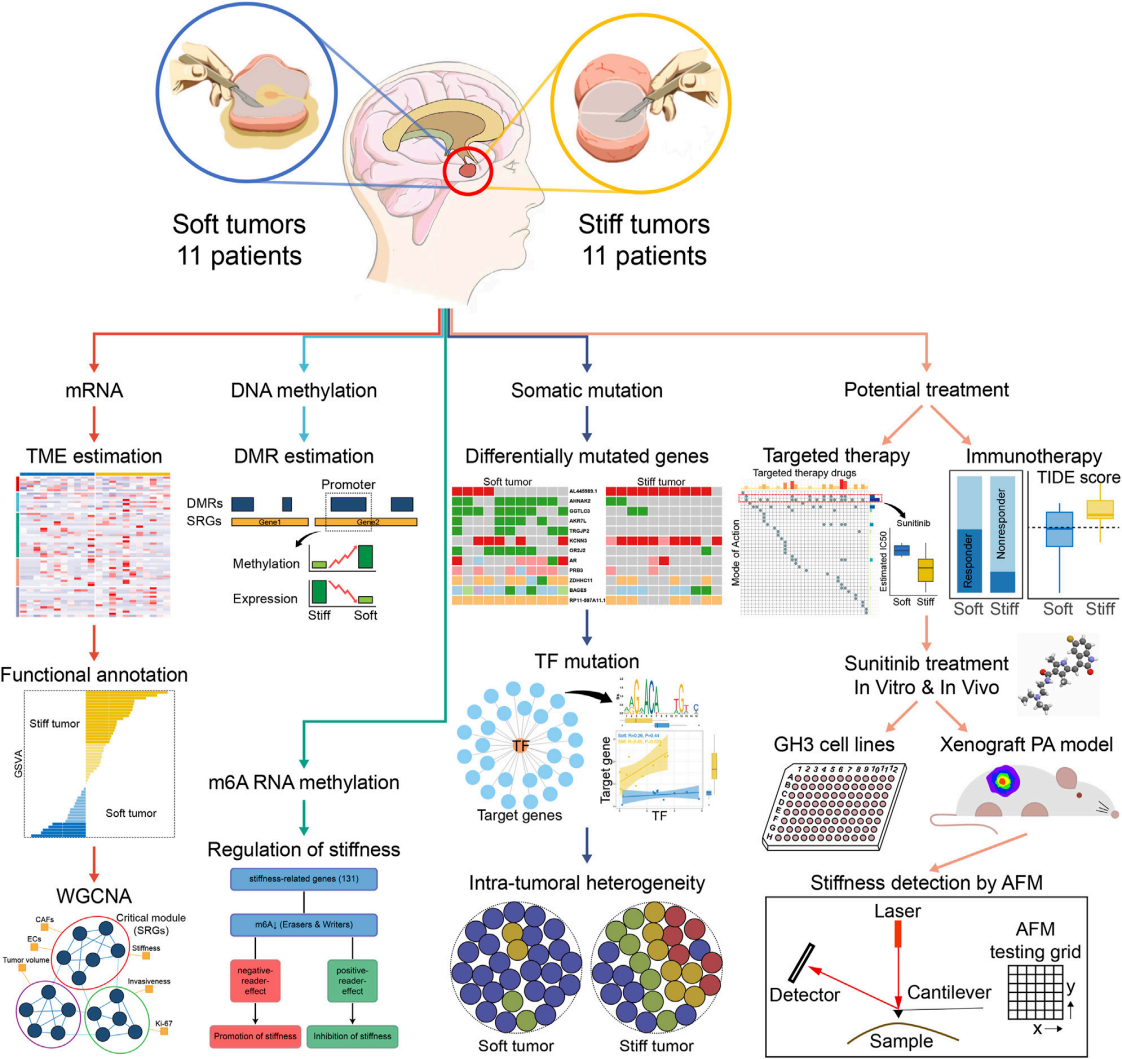
The overall workflow of the integrated transcriptomic, genomic, and epigenomic profiling analyses in this study.

**FIGURE 2:**
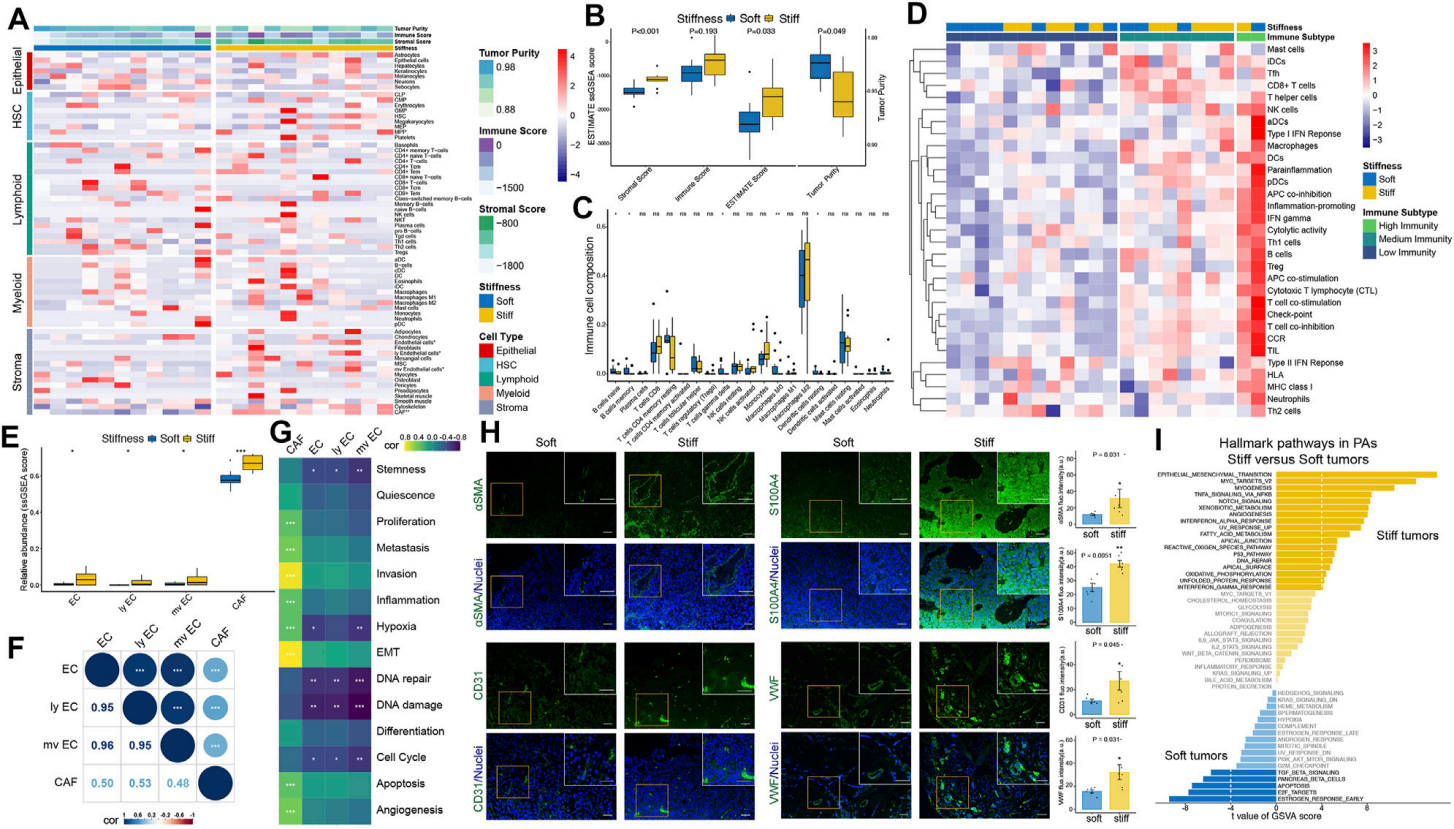
Estimation of tumor immune microenvironment patterns associated with the stiffness of PAs. **(A)** Heatmap illustrating the cellular components of PA samples, including epithelial, hematopoietic progenitor, lymphoid, myeloid, and stromal cell clusters, quantified by the xCell algorithm. **(B)** Comparisons of stromal score, immune score, ESTIMATE score and tumor purity between soft and stiff tumors. **(C)** Comparisons of the abundances of 22 immune cells between soft and stiff tumors. **(D)** Heatmap illustrating the immune subtypes of PA patients, which were categorized based on the overall immune activity of tumors. **(E)** Comparisons of ECs, lymphatic ECs, microvascular ECs, and CAFs between soft and stiff tumors. **(F)** Correlation analysis of ECs, lymphatic ECs, microvascular ECs, and CAFs. **(G)** Correlation analysis of ECs, CAFs and 14 functional processes of cancer cells. **(H) Left panels:** Immunofluorescence staining of CAF markers (αSMA and S100A4) and EC markers (CD31 and VWF) (green) in soft and stiff tumors. Cell nuclei were counterstained with DAPI (blue). Scale bar: 50 μm. **Right panels:** Quantification of the fluorescence intensity of four cell markers between soft and stiff PAs. a. u., arbitrary unit. **(I)** The differential hallmark pathways associated with stiff and soft tumors. * means *p* < 0.05, ** means *p* < 0.01, and *** means *p* < 0.001.

The overall workflow of this integrated transcriptomic, genomic, and epigenomic profiling analysis is displayed in Figure 1. First, the xCell algorithm was employed to quantify the cellular components of PA samples, including epithelial, hematopoietic progenitor, lymphoid, myeloid, and stromal cell clusters (Figure 2A). Then, the ESTIMATE algorithm was employed to assess the overall TME of PAs. Compared with the respective scores in soft tumors, the stromal and ESTIMATE scores were significantly higher and the tumor purity score was significantly lower in stiff PAs (all *p* < 0.05), and stiff PAs demonstrated high abundances of general stromal cells and low tumor purity (Figure 2B). However, the immune score did not differ significantly between soft and stiff tumors, and the CIBERSORT algorithm also demonstrated no significant difference in the infiltration abundances of the majority of TIICs between the two groups (Figure 2C). Next, the enrichment levels of 31 immune signatures, representing the overall immune activity of PAs, were quantified by ssGSEA, and the 22 PA patients were classified into three immune subtypes by unsupervised hierarchical clustering (Figure 2D). The distributions of soft and stiff tumors in the high-, medium-, and low-immunity groups did not differ significantly (*p* = 0.659).

Among all the cellular components of PA samples, general ECs, lymphatic ECs, microvascular ECs, and CAFs showed significantly higher abundances in stiff tumors than in soft tumors (Figure 2E), and they were highly positively correlated (Figure 2F). Furthermore, to elucidate the functional associations between ECs, CAFs and 14 functional processes of cancer cells, Pearson correlation analysis was used, and the results demonstrated that general, lymphatic, and microvascular ECs were negatively correlated with the cell cycle, DNA damage/repair, hypoxia, and stemness, whereas CAFs were positively correlated with angiogenesis, apoptosis, epithelial-mesenchymal transition (EMT), hypoxia, inflammation, invasion, metastasis, and proliferation (Figure 2G). To verify the difference in stromal cells between soft and stiff tumors, we performed immunofluorescence staining of CAF markers (αSMA and S100A4) and EC markers (CD31 and VWF) (Figure 2H). Significantly higher expression levels of αSMA, S100A4, CD31 and VWF were observed in stiff tumors than in soft tumors, suggesting that there are higher abundances of CAFs and ECs in stiff PAs.

GSVA was further performed to explore the hallmark pathways and underlying molecular mechanisms associated with the stiffness of PA samples. A total of 23 differentially enriched molecular pathways were identified, including 18 pathways positively correlated with stiff tumors and five pathways positively correlated with soft tumors (Figure 2I). EMT was the most significant hallmark pathway related to stiff PAs, suggesting a potential molecular mechanism underlying the stiff phenotype.

All these findings suggest that stromal cells, especially ECs and CAFs, and EMT might play critical roles in contributing and promoting the stiffening of PAs, whereas immune cells might not be associated with the stiffness of PAs.

### Identification of the Top Stiffness-Related Gene Module of PAs by WGCNA

**FIGURE 3:**
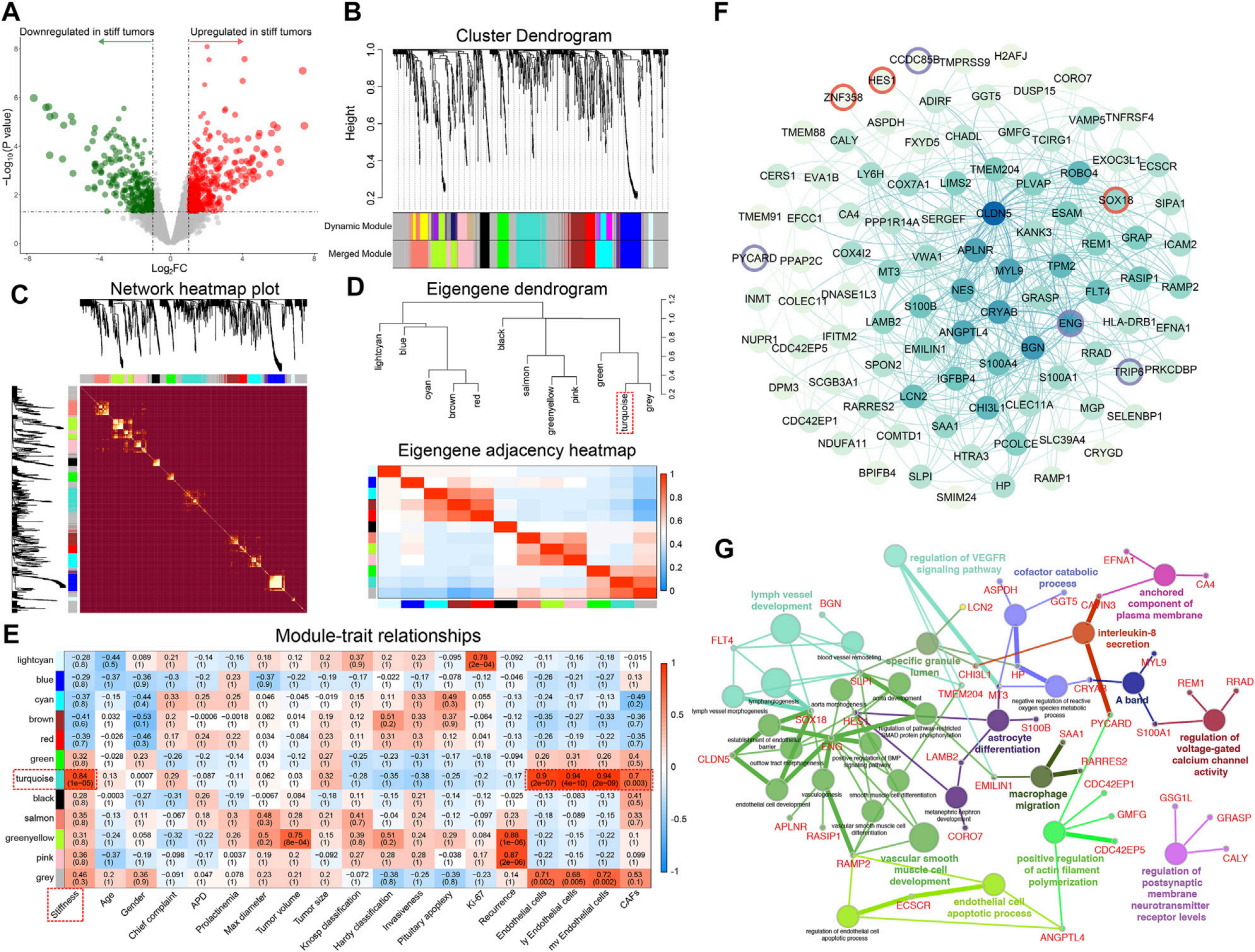
Identification of the top stiffness-related gene module of PAs by WGCNA. **(A)** Volcano plot displaying the DEGs between stiff and soft tumors. Red dots represent the upregulated genes, and green dots represent the downregulated genes in the stiff PAs. **(B)** Cluster dendrogram of the DEGs. **Upper panel:** Each branch represents one single gene. **Lower panel:** Each color represents one coexpression module. **(C)** Heatmap illustrating the interactions of coexpressed genes. The brightness of yellow in the heatmap represents the degree of connectivity of different modules, with a lighter color indicating greater overlap. The colors of the horizontal and vertical axes represent different modules, and the branches represent different genes. **(D) Upper panel:** Hierarchical clustering of module genes. **Lower panel:** Heatmap of the adjacencies in the module gene network. **(E)** Heatmap displaying the correlations between module eigengenes and the clinical traits of patients with PA. The turquoise module was the most critical module and was positively correlated with stiffness, general ECs, lymphatic ECs, microvascular ECs, and CAFs. **(F)** PPI network including 131 SRGs from the turquoise module. A change in dot color from lighter to darker indicates an increasing degree of gene connectivity. Red circles represent TFs, and purple circles represent TF cofactors. **(G)** Functional enrichment analysis of the SRGs. Different dot colors indicate different GO clusters.

A total of 1,288 DEGs between soft and stiff PAs were identified, including 759 upregulated and 529 downregulated genes in stiff tumors, and the results are displayed in the volcano plot (Figure 3A). To determine the phenotypic relevance of the DEGs, WGCNA was performed to identify the most critical gene module related to the stiffness of tumors. By using the pickSoftThreshold method, 30 was selected as the soft-thresholding power needed to achieve a scale-free topology fit index >0.85 and maintain optimal mean connectivity (Supplementary Figure S1). A hierarchical clustering dendrogram was obtained, and 12 gene modules were ultimately generated by employing the Dynamic Tree Cut package (Figure 3B). The gray module, including all the genes that could not be enrolled into any other modules, was excluded from the subsequent analysis. The network heatmap demonstrated that MEs were highly correlated within each module, suggesting that highly coexpressed eigengenes in the same module (Supplementary Table S2) may possess similar biological significance and function together (Figure 3C). In addition, to explore the coexpression similarity of all modules, the modules were mainly divided into two clusters in the hierarchical clustering dendrogram and the eigengene adjacency heatmap according to their correlations with each other (Figure 3D). Furthermore, analyses of the correlations between MEs and clinical traits were performed, and the turquoise module, consisting of 131 genes, was positively correlated with stiffness (R = 0.84, *p* = 1 × 10^−05^), general ECs (R = 0.90, *p* = 2 × 10^−07^), lymphatic ECs (R = 0.94, *p* = 4 × 10^−10^), microvascular ECs (R = 0.94, *p* = 2 × 10^−09^), and CAFs (R = 0.70, *p* = 0.003) (Figure 3E). There were high correlations of MM for genes in the turquoise module and GS with stiffness, ECs and CAFs, which also indicated the critical roles of the turquoise module in promoting the stiffness of PAs (Supplementary Figure S2). Hence, the 131 MEs in the turquoise module were defined as SRGs.

To better illustrate the functional associations and topology of the interactions between SRGs, we constructed a PPI network (Figure 3F). Three TFs (SOX18, HES1, and ZNF358) and 4 TF cofactors (CCDC85B, ENG, PYCARD, and TRIP6) were identified in the network. Functional annotations of the 131 SRGs indicated that they were mainly enriched in 14 GO clusters, representing 30 GO terms (Figure 3G). Vascular smooth muscle cell development, including 11 terms (36.7%), was the most important functional cluster, followed by lymph vessel development (13.3%), endothelial cell apoptotic process (6.7%), regulation of VEGFR signaling pathway (6.7%), etc. These findings suggest that the SRGs might regulate the stiffness of pituitary tumors by regulating the development, differentiation, and apoptosis of ECs and CAFs and related molecular pathways.

### Associations Between DMRs and the Stiffness of PAs

**FIGURE 4:**
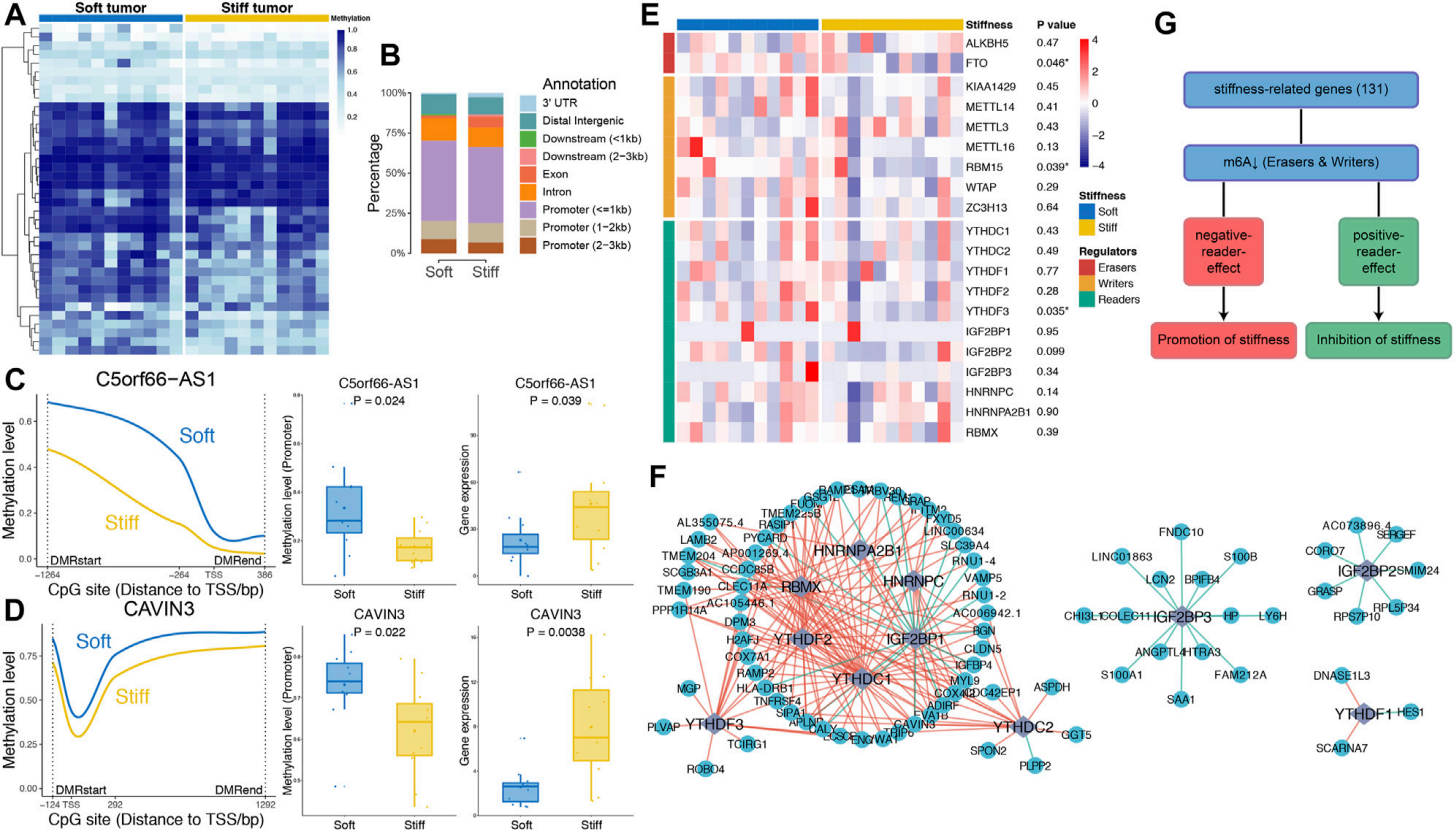
Associations of DNA methylation and m6A RNA methylation with the stiffness of PAs. **(A)** Heatmap illustrating the 38 DMRs between soft and stiff tumors. **(B)** Comparisons of hypermethylated DMRs between soft and stiff tumors. **(C,D) Left panel:** Comparisons of the methylation levels of CpG sites within the DMRs of promoter regions between soft and stiff PAs. **Middle panel:** Comparisons of the methylation levels of promoter regions between soft and stiff PAs. **Right panel:** Comparisons of the gene expression levels between soft and stiff PAs. **(E)** Heatmap comparing 20 m6A regulators between soft and stiff tumors. **(F)** Analysis of the correlation between reader and SRG expression. Blue dots represent SRGs, and purple rhombi represent readers. Red lines represent negative correlations, and green lines represent positive correlations. **(G)** The overall role of m6A in regulating the stiffness of PAs is believed to be two-sided, including promotion and inhibition of stiffness.

To investigate epigenomic associations with the stiffness of PAs, genome-wide methylation profiles were utilized to determine DMRs between soft and stiff tumors. A total of 188 significant CpG sites and 38 DMRs were identified, including 25 regions with higher methylation in soft tumors and 13 regions with higher methylation in stiff tumors (Figure 4A and Supplementary Table S3). The hypermethylated DMRs in stiff tumors were more enriched within exons (*p* = 0.037) than were those in soft tumors, whereas the percentage of hypermethylated DMRs in the promoter regions did not differ significantly between the two groups (Figure 4B). DMR-associated genes were identified for all the DMRs, and only two of them (C5orf66-AS1 and CAVIN3) belonged to the SRGs. The methylation levels of the promoter regions of C5orf66-AS1 and CAVIN3 were significantly lower in stiff tumors than in soft tumors, and in contrast, their gene expression was higher in the stiff tumors (Figures 4C,D). The strong negative correlations between promoter methylation and mRNA expression levels of C5orf66-AS1 and CAVIN3 suggest that aberrant DNA methylation of the SRGs might be a crucial process contributing to the stiffening of PAs.

### Dynamic Bilateral Regulation of Stiffness by m6A

To explore the potential roles of RNA modifications in regulating the stiffness of PAs, we assessed the associations between m6A regulators and SRGs. Twenty m6A regulators were compared between soft and stiff tumors, and we found that FTO and RBM15 were significantly downregulated in stiff tumors, suggesting low m6A levels in these PAs (Figure 4E). Analysis of the correlation between reader and SRG expression demonstrated that the post-methylation regulatory effects on target genes were mainly negative, but some readers showed positive effects (IGF2BP1 and IGF2BP3) (Figure 4F). m6A has been previously reported to have a dual role in regulating the stiffness of PAs, mostly promoting stiffening and rarely inhibiting stiffening of pituitary tumors (Figure 4G).

### High Intratumoral Heterogeneity in Stiff PAs

**FIGURE 5:**
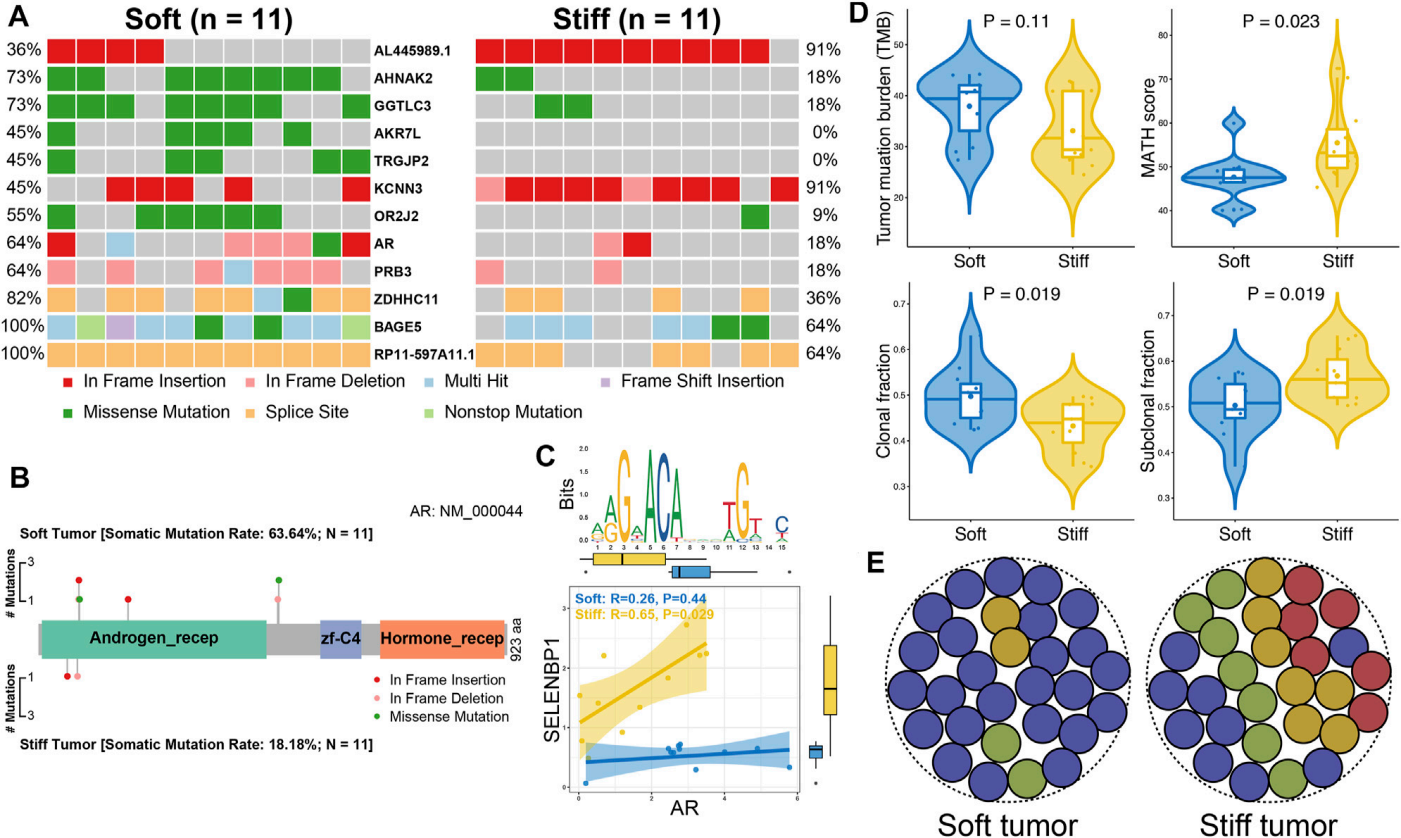
Comparisons of somatic variations between soft and stiff tumors. **(A)** Waterfall plots showing the mutation types and frequencies of 12 DMGs between soft and stiff tumors. **(B)** Lollipop plot illustrating the mutation sites and types of AR in soft and stiff PAs. aa: amino acid. Androgen_recep: androgen receptor (6-449 aa); zf-C4: zinc-finger, C4 type (two domains) (558-626 aa); Hormone_recep: ligand-binding domain of nuclear hormone receptor (692-877 aa). **(C)** Analysis of the correlation between AR and its target gene (SELENBP1) in soft and stiff PAs. An AR motif is displayed in the upper panel. **(D)** Comparisons of TMB, MATH score, clonal fraction, and subclonal fraction between soft and stiff tumors. **(E)** Schematic diagram displaying the intratumoral heterogeneity of soft and stiff PAs.

**FIGURE 6:**
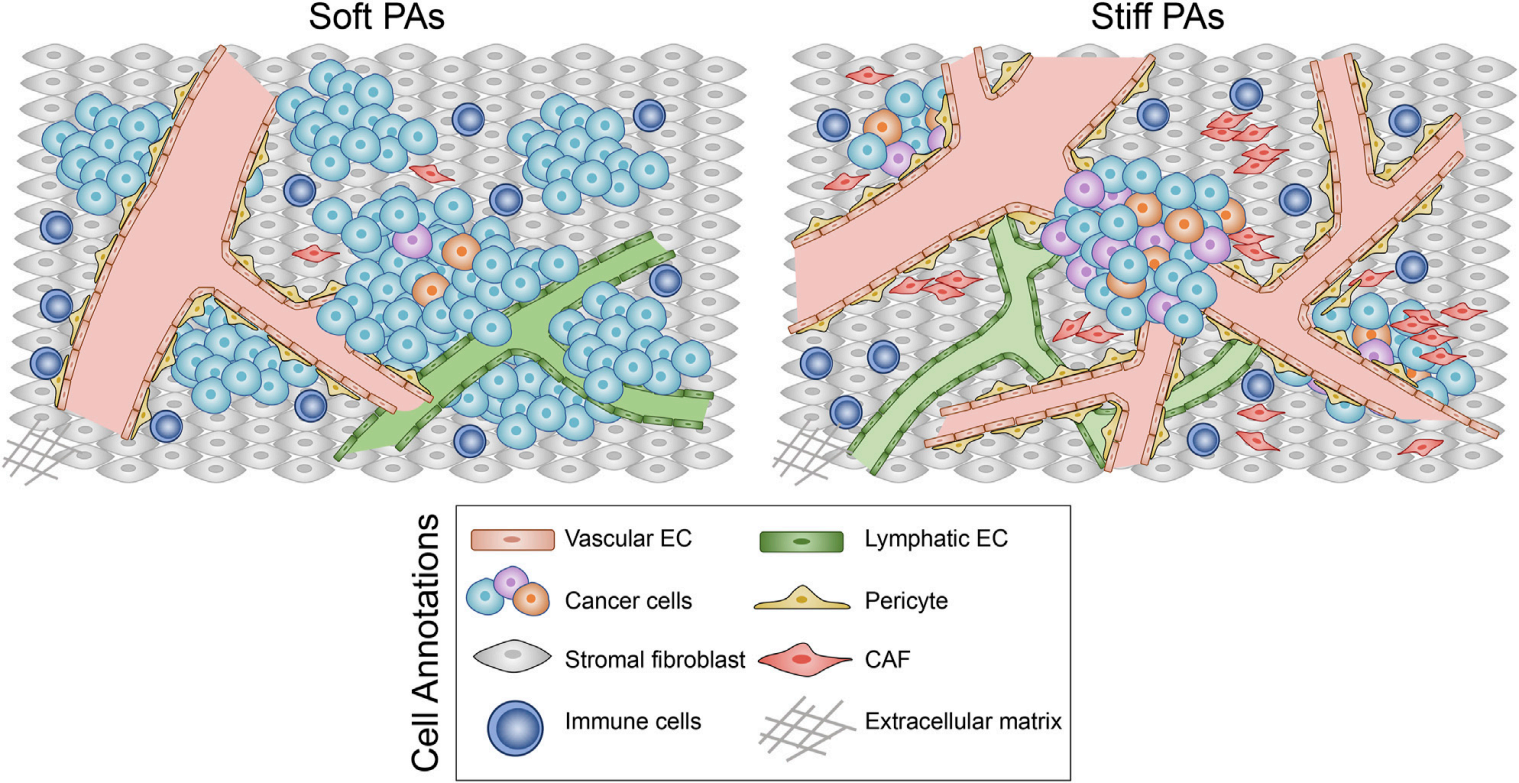
Schematic diagram illustrating the general TME patterns of soft and stiff tumors.

Somatic mutation analysis was performed to investigate distinct genomic alterations between soft and stiff tumors that have been reported to regulate the TME and immunity. A total of 12 DMGs were identified between the two groups (Figure 5A), and the mutation frequency of AR, the only TF, was significantly higher in the soft tumor group than in the stiff tumor group (63.6 vs. 18.2%, *p* = 0.035). A lollipop plot was generated and illustrated that the mutation sites and types of AR were distinct in the soft and stiff tumors (Figure 5B). TF mutation has been reported to play a crucial role in pathogenesis; hence, we performed correlation analysis between AR and its target genes. SELENBP1, one of the SRGs and one of the target genes of AR, was positively correlated with stiff tumors (R = 0.65, *p* = 0.029) (Figure 5C). In addition, as shown in Figure 5D, patients with stiff tumors had lower TMB (*p* = 0.11), higher MATH score (*p* = 0.023), lower clonal fraction (*p* = 0.019), and higher subclonal fraction (*p* = 0.019) than patients with soft tumors, indicating that there is higher intratumoral heterogeneity in stiff PAs (Figure 5E). All these findings suggest underlying differences in immune evasion and immunotherapy response between soft and stiff tumors. The general TME patterns of soft and stiff tumors are displayed in Figure 6.

### Identification of Potential Drugs for Treating Stiff PAs

**FIGURE 7:**
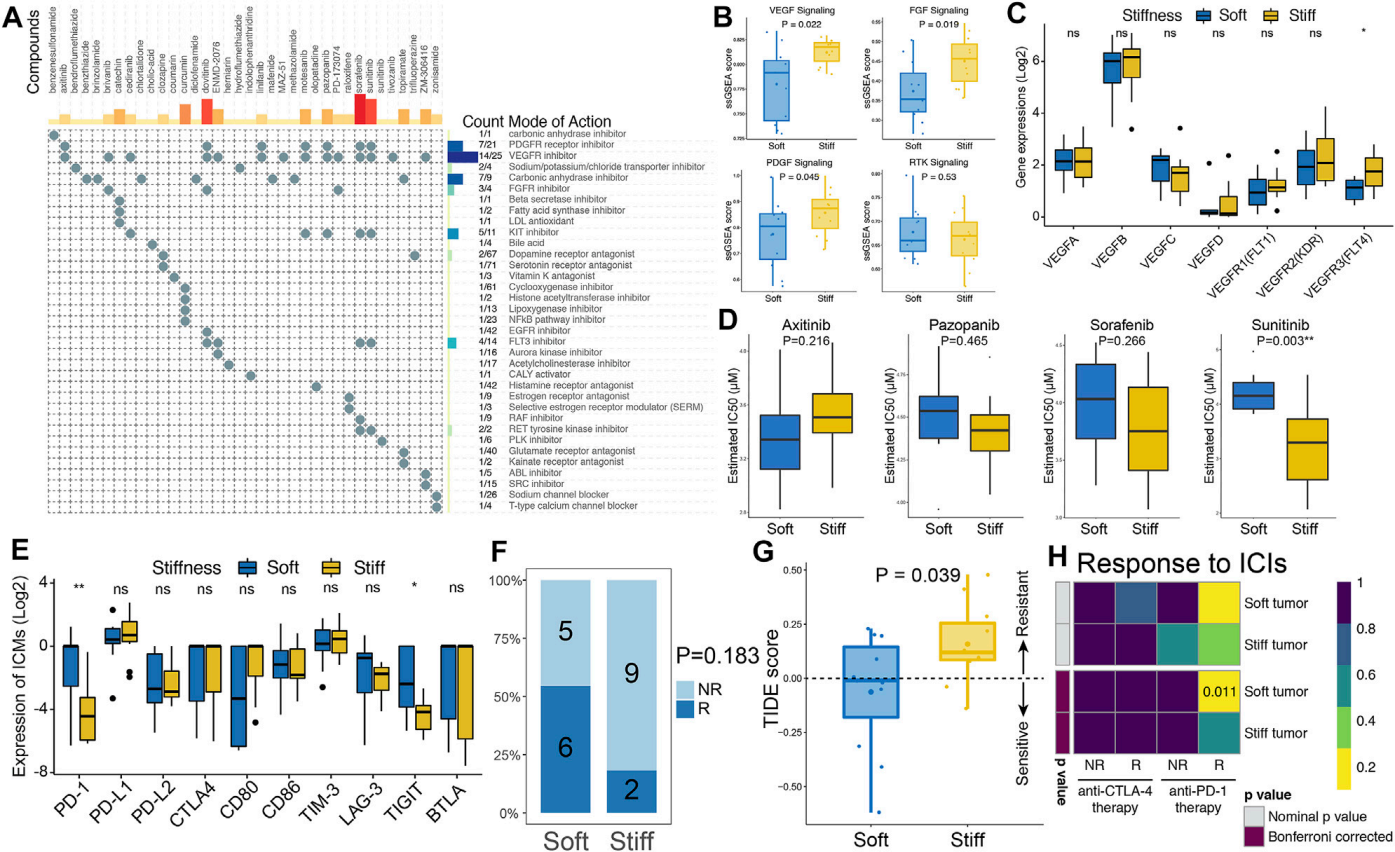
Prediction of PA targeted therapy response. **(A)** CMap-based exploration of candidate drugs and molecular pathways that can be used for treatment of stiff PAs based on the SRGs. The MoA analysis revealed 35 molecular pathways targeted by 36 compounds. **(B)** Comparisons of the ssGSEA enrichment scores of the VEGF, PDGF, FGF and RTK signaling pathways between soft and stiff tumors. **(C)** Comparisons of the gene expression of structurally related factors and receptors of the VEGF family between soft and stiff tumors. **(D)** Comparisons of the IC50 values of axitinib, pazopanib, sorafenib, and sunitinib as treatment between soft and stiff PA samples, as estimated by the pRRophetic algorithm based on GDSC data. **(E)** Comparisons of the gene expression of ICMs between soft and stiff tumors. **(F)** Comparisons of the proportions of nonresponders and responders to immunotherapy between the soft and stiff tumor groups. NR, nonresponder; R, responder. **(G)** Comparisons of the TIDE score between soft and stiff tumors. A TIDE score <0 indicates sensitivity to immunotherapy, and a TIDE score >0 indicates resistance to immunotherapy. **(H)** Subclass mapping analysis for predicting the likelihood of response to ICI treatments in soft and stiff PAs.

CMap analysis of the SRGs and related molecular pathways was performed to explore potential compounds that could be used to treat stiff PAs. MoA analysis revealed 35 molecular pathways targeted by 36 compounds in the stiff tumors (Figure 7A and Supplementary Table S4). Regarding the most enriched and critical MoAs, there were 14 compounds sharing the same MoA as VEGFR inhibitors, 7 compounds sharing the same MoA as PDGFR inhibitors, and three compounds sharing the same MoA as FGFR inhibitors in the stiff tumors. Hence, the VEGF, PDGF and FGF signaling pathways might serve as potential therapeutic targets for stiff PAs. As shown in Figure 7B, the enrichment levels of the VEGF, PDGF and FGF signaling pathways were significantly higher in the stiff tumors than in the soft tumors, indicating that there is activation of these molecular pathways in stiff PAs and that they have potential roles in promoting the stiffening of PAs. Regarding the structurally related factors and receptors of the VEGF family, only VEGFR3 (FLT4) was observed to be significantly higher in the stiff PAs (Figure 7C). In addition, the therapeutic responses of stiff PAs to axitinib, pazopanib, sorafenib, and sunitinib were evaluated by using the pRRophetic algorithm based on GDSC data. By integrating the gene expression profiles of cell lines and PA samples, we estimated the IC50 values of the four drugs in each PA patient using ridge regression analysis. The estimated IC50 value of sunitinib was significantly lower in stiff PAs than in soft PAs (*p* = 0.003), indicating that PA patients with stiff tumors tended to be more sensitive to sunitinib therapy (Figure 7D).

### Stiff PAs Were More Resistant to Immunotherapy

Regarding immune checkpoint molecules (ICMs), the expression levels of PD-1 and TIGIT were significantly higher in the soft PAs than in the stiff PAs, whereas other ICMs did not differ between the two groups (Figure 7E). Then, the TIDE algorithm, which quantifies T cell dysfunction signatures, was applied to predict the likelihood of immunotherapy response in PA patients. The proportion of responders to immunotherapy in the patients with soft PAs was two times greater than that in the stiff tumors (54.5 vs. 18.2%, *p* = 0.183) (Figure 7F). The TIDE scores, calculated based on gene expression profiles, were significantly higher in the stiff tumors than in the soft tumors (*p* = 0.039), suggesting that the patients with soft PAs were more sensitive to immunotherapy than were those with stiff tumors (Figure 7G). In addition, an unsupervised subclass mapping method was utilized to predict the clinical response to ICIs, including PD1 and CTLA4 inhibitors, of soft and stiff tumors. Patients with soft tumors were more likely to respond to anti-PD-1 therapy than those with stiff tumors (FDR = 0.011), whereas neither group was more sensitive to anti-CTLA4 therapy (Figure 7H). Generally, the higher TMB, lower intratumoral genetic heterogeneity, lesser subclonality, and higher ICM expression of soft PAs might explain why they are more sensitive to immunotherapy, especially anti-PD-1 treatment, than stiff PAs.

### Sunitinib Inhibited PA Growth *in Vitro* and *in Vivo*, and Reduced Tumor Stiffness in Xenograft PA Models Detected by AFM

**FIGURE 8:**
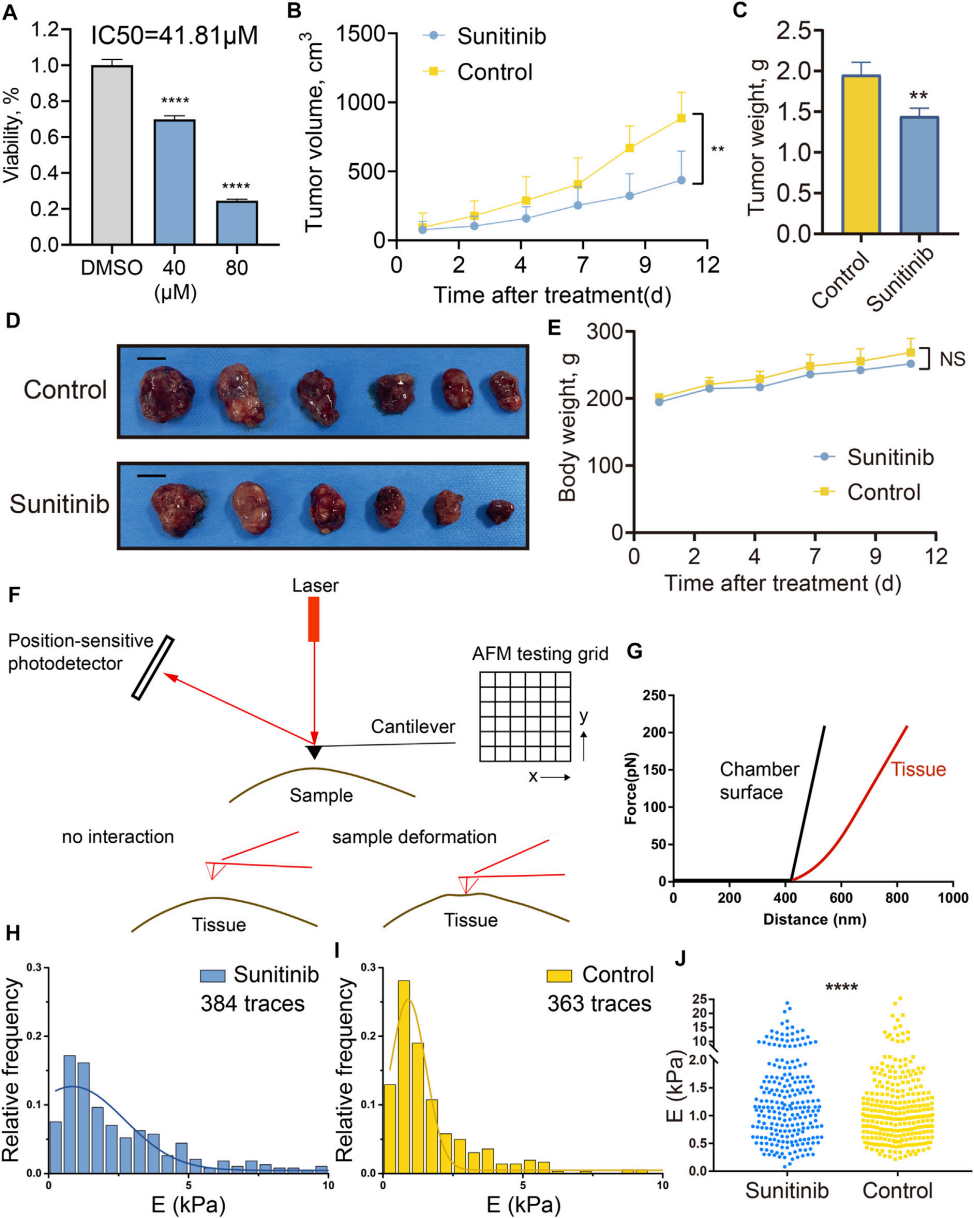
Sunitinib inhibited PA growth *in vitro* and *in vivo*, and reduced tumor stiffness in xenograft PA models examined by AFM. **(A)** Cell viability of GH3 cells treated with sunitinib or DMSO for 2 days. Three independent experiments were repeated for each result. *** means *p* < 0.001. Bar represents mean ± SD. **(B)** Tumor volume of rats treated with sunitinib or vehicle control once daily, measured for 12 days. **(C)** Tumor weight of rats treated with sunitinib or vehicle control. ** means *p* < 0. 01. **(D)** Macroscopic image of the resected tumors. Scale bar: 1 cm. **(E)** Body weight of rats measured for 12 days “NS” means no significant difference between groups. **(F)** Working schematic of the AFM setup used for mechanical property measurement of tumor samples. **(G)** Slope changes deliver Young’s modulus changes of different interaction surfaces. Black curve represents force vs. distance curve recorded for the chamber surface (reference), and the red one represents force vs. distance curve for the tumor tissue. Statistic histograms show the distributions of Young’s modulus of all traces recorded for tumor tissues in the sunitinib treatment group **(H)** and control group **(I)**. Mean Young’s modulus (xc ± SE), calculated by the Gaussian fitting, was 0.85 ± 0.34 kPa for the sunitinib treatment group and 0.90 ± 0.03 kPa for the control group. E (kPa) means the Young’s modulus of the traces in each sample. **(J)** Comparisons of the Young’s modulus of all collected traces between two groups. **** means *p* value <0.0001 via using the Kolmogorov-Smirnov test.

To further investigate the sensitivity of PAs to sunitinib treatment, we tested the cell viability of GH3 cell line treated by sunitinib. After 2 days of treatment, sunitinib exhibited promising anti-proliferative effects in GH3 cells, with a half-maximal inhibitory concentration value of 41.81 µM (Figure 8A). Subsequently, to evaluate the impact of the sunitinib on GH3 cells *in vivo*, we generated a xenograft PA model by transplanting GH3 cells subcutaneously into the flanks of Wistar Furth rats. Once the tumor volume approached approximately 100 mm^3^, treatment was started with intragastric administration of 40 mg/kg sunitinib or vehicle control once daily for 12 days. All rats were sacrificed after completion of the 12-days experiment. Sunibtinib treatment significantly inhibited tumor growth with regard to tumor volume (59% inhibition, *p* < 0.05) and tumor weight (37% inhibition, *p* < 0.05) in comparison with the control regimen (Figures 8B–D). Additionally, sunibtinib treatment showed minimal effects on rats’ body weights, demonstrating its safety (Figure 8E). Then, AFM was further applied to examine the mechanical properties of the resected tumor samples from two groups. The overall working schematic of the AFM setup used for mechanical property measurement, especially Young’s modulus changes, was shown in Figures 8F,G. The distributions of Young’s modulus of all traces recorded for the tumors in two groups were displayed by the statistic histograms (Figures 8H,I). Mean Young’s modulus (xc ± SE), calculated by the Gaussian fitting, was 0.85 ± 0.34 kPa for the sunitinib treatment group and 0.90 ± 0.03 kPa for the control group. Kolmogorov-Smirnov test demonstrated that the Young’s modulus of collected traces was lower in the sunitinib treatment group than that in the control group (*p* < 0.0001; Figure 8J). All these findings indicated that sunitinib can inhibit tumorigenesis and reduce the stiffness of pituitary tumors, which suggested sunitinib could serve as a potential candidate drug for stiff PAs.

## Discussion

As the first-line and even the only therapy for PAs, total resection of tumors in the transsphenoidal surgery was extremely important for curing PAs. Despite rapid progression of endoscopic surgery systems, tumor stiffness has become the most critical factor that affects the surgical resection rate in invasive tumors. Particularly, total resection of stiff PAs invading the cavernous sinus is the most challenging for neurosurgeons (Bao et al., 2016; Kim et al., 2018). Consistent with this, in this study, stiff tumors had a significantly lower gross total resection rates than soft tumors, and the recurrence rate of stiff tumors was higher. These results suggest that the basis of this investigation is well founded and that the samples used in the study are robust. However, the molecular mechanisms that regulate the mechanical properties and contribute to stiffening in PAs were still unknown.

Tissue stiffness can be increased by collagen content and fiber organization, which lead to angiogenesis (Bayer et al., 2019; Shen et al., 2020). In our study, the xCell algorithm revealed that ECs and CAFs were more abundant in stiff tumors than in soft tumors. EMT might play critical roles in the stiffening of PAs. Furthermore, immunofluorescence staining of CAF markers (αSMA and S100A4) and EC markers (CD31 and VWF) verified the differences in stromal cells. Therefore, CAFs and ECs play important roles in tumor stiffness. In addition, the SRGs identified by the transcriptome analysis were enriched in EC and CAF regulation pathways. These results imply that these SRGs probably lead to a high abundance of ECs and CAFs, which might lead to stiffening of tumors.

DNA methylation, in which alterations of CpG dinucleotides block the transcriptional mechanism and silence gene expression, is the most frequently studied epigenetic phenomenon (Pease et al., 2013). By studying the correlation between DNA methylation data with SRG expression data, we found that the DNA methylation level was negatively correlated with the RNA expression of SRGs, including C5orf66-AS1 and CAVIN3, which indicates that SRGs are probably regulated by DNA methylation. C5orf66-AS1 is a long noncoding RNA (lncRNA) that suppresses the development and invasion of pituitary null cell adenomas (Yu et al., 2017). Two groups also reported aberrant methylation-mediated downregulation of the lncRNA C5orf66-AS1. One study was in gastric cardia adenocarcinoma (Guo et al., 2018), and the other study was in head and neck squamous cell carcinoma (Sailer et al., 2018). The relationships between DNA methylation, SRGs, and ECs and CAFs are probably responsible for the regulation of PA stiffening.

RNA methylation is an important regulatory factor in different physiological and pathological processes. including development (Yoon et al., 2017; Li et al., 2018; Ma et al., 2018; Wang et al., 2018), neurogenesis (Li et al., 2017), innate immunity (Zheng et al., 2017), and tumorigenesis (Zhang et al., 2016; Zhang et al., 2017a; Cui et al., 2017; Ma et al., 2017; Chen et al., 2018; Chen et al., 2019; Han et al., 2019; Lan et al., 2019; Niu et al., 2019; Jin et al., 2020). m6A has been shown to play a role in fate determination of cells (Batista et al., 2014; Aguilo et al., 2015; Chen et al., 2015; Zhang et al., 2017b). In this study, we found that m6A regulators (FTO and RBM15) were significantly downregulated in stiff tumors, suggesting aberrant m6A levels in stiff PAs. Moreover, the expression levels of m6A “readers” and SRGs were mainly decreased, while some readers showed increased expression. m6A has been shown to regulate RNA expression levels by influencing RNA degradation (Wang et al., 2014; Ke et al., 2017). Therefore, m6A might play a role in the posttranscriptional regulation of SRGs and further regulate EC and CAF production. Consistent with this, m6A was found to be critical for the development of cardiac fibrosis (Li et al., 2021). In another study, the m6A-mediated MALAT1/miR-145/FAK pathway was found to be involved in renal fibrosis (Liu et al., 2020). Three ECM components (COL6A1, LAMA5, and FN1) are target genes of the m6A reader IGF2BP3 and can be regulated in an m6A-dependent manner (Gu et al., 2020). Together, these mechanisms provide strong evidence that m6A regulation might play an important role in ECM component production, which is important for tissue stiffening.

Consistent with the increased levels of ECM components seen in stiff tumors, tumor heterogeneity is also increased in stiff tumors. Compared with soft tumors, stiff tumors showed higher MATH scores, indicating that there is higher intratumoral heterogeneity in stiff PAs. Increased heterogeneity can lead to tumor evolution (McGranahan and Swanton, 2017; Pelham et al., 2020), drug resistance (Lim and Ma, 2019), and immune evasion (Vinay et al., 2015). In addition, immune markers were decreased in stiff tumors, and stiff PAs were likely to show a decreased response to immunotherapy. Taken together, these data suggest that stiff tumors have higher heterogeneity, greater subclonality, and fewer immunotherapy targets than soft tumors, which make them harder to treat with targeted therapy. Therefore, transforming stiff tumors into soft tumors can make tumor treatment easier.

We used CMap to obtain compounds that can inhibit SRG expressions. MoA analysis revealed compounds targeting the most enriched and critical molecular pathways. Sunitinib was ultimately selected as the drug to which stiff PAs would be most sensitive using the pRRophetic algorithm based on GDSC database. In the GH3 cell cultures, sunitinib significantly decrease the cell viability. In addition, since the pituitary gland sites outside the blood-brain barrier, a rat xenograft flank model was applied to explore the efficacy of sunitinib on PA *in vivo*. Sunitinib significantly inhibited GH tumorigenesis but did not show toxicity to rats when orally administered for 12 days. In addition, AFM-based Young’s modulus measurement also demonstrated the stiffness of PA samples were significantly reduced after sunitinib treatment, which suggested sunitinib could serve as a potential candidate drug for stiff PAs. As reported in the literature, sunitinib was developed to inhibit growth factor receptor tyrosine kinases in ECs and pericytes, which are implicated in angiogenesis (Tran et al., 2016). Sunitinib is a standard-of-care first-line therapy for patients with advanced renal cell carcinoma (Motzer et al., 2019). Sunitinib was also reported to block survival in the rat pituitary cell line GH4C (Chenlo et al., 2019). In summary, sunitinib is a potential therapeutic agent for stiff PAs. Prospective clinical studies investigating sunitinib will be necessary to assess its efficacy.

In conclusion, we assessed the molecular landscape during PA stiffening and discovered unique features of cell components and gene regulation in stiff tumors. Our results indicate that the epigenome and epitranscriptome are essential in the regulation of tumor stiffness-related RNA expression. *In vitro* and *in vivo* also provided evidence that sunitinib has the potential to reverse PA stiffening. Therefore, characterizing the mechanism by which PAs become stiff will offer a new theoretical basis for developing novel therapies for individualized treatment.

## Data Availability

The datasets presented in this study can be found in online repositories. The names of the repository/repositories and accession number(s) can be found below: https://ngdc.cncb.ac.cn/gsa-human/, HRA000954, HRA000955, and HRA000956.

## Supplementary Material

Supplementary Figure S1 Clustering of PA samples and determination of soft-thresholding power. **(A)** The hierarchical clustering dendrogram of PA samples. **(B)** Analysis of the scale-free fit index for various soft-thresholding powers. The red line indicates a correlation coefficient of 0.85. **(C)** Analysis of the mean connectivity for various soft-thresholding powers.

Supplementary Figure S2 Scatter plots of Module Membership (x-axis) in the turquoise module vs. Gene Significance for stiffness **(A)**, general ECs **(B)**, lymphatic ECs **(C)**, microvascular ECs **(D)**, and CAFs **(E)** (y-axis).
