# Supplementary material for: Multi-Omics Investigations Revealed Underlying Molecular Mechanisms Associated With Tumor Stiffness and Identified Sunitinib as a Potential Therapy for Reducing Stiffness in Pituitary Adenomas
Source: Front Cell Dev Biol. 2022 Mar 15;10:820562. doi: 10.3389/fcell.2022.820562 (PMC8965615; doi:10.3389/fcell.2022.820562)
Supplement: Supplementary file 1 [file Table1.DOCX]

**Supplementary Table 1.** The gene signature profiles for overall immune activity and functional states of PA cells.

| Gene signatures | | Gene symbols |
| --- | --- | --- |
| Immune signatures | aDCs | CD83, LAMP3, CCL1 |
|  | APC co-inhibition | C10orf54, CD274, LGALS9, , PDCD1LG2, PVRL3 |
|  | APC co-stimulation | CD40, CD58, CD70, ICOSLG, SLAMF1, TNFSF14, TNFSF15, TNFSF18, TNFSF4, TNFSF8, TNFSF9 |
|  | B cells | BACH2, BANK1, BLK, BTLA, CD79A, CD79B, FCRL1, FCRL3, HVCN1, RALGPS2 |
|  | CCR | CCL16, TPO, TGFBR2, CXCL2, CCL14, TGFBR3, IL11RA, CCL11, IL4I1, IL33, CXCL12, CXCL10, BMPER, BMP8A, CXCL11, IL21R, IL17B, TNFRSF9, ILF2, CX3CR1, CCR8, TNFSF12, CSF3, TNFSF4, BMP3, CX3CL1, BMP5, CXCR2, TNFRSF10D, BMP2, CXCL14, CCL28, CXCL3, BMP6, CCL21, CXCL9, CCL23, IL6, TNFRSF18, IL17RD, IL17D, IL27, CCL7, IL1R1, CXCR4, CXCR2P1, TGFB1I1, IFNGR1, IL9R, IL1RAPL1, IL11, CSF1, IL20RA, IL25, TNFRSF4, IL18, ILF3, CCL20, TNFRSF12A, IL6ST, CXCL13, IL12B, TNFRSF8, IL6R, BMPR2, IFNE, IL1RAPL2, IL3RA, BMP4, CCL24, TNFSF13B, CCR4, IL2RA, IL32, TNFRSF10C, IL22RA1, BMPR1A, CXCR5, CXCR3, IFNA8, IL17REL, IFNB1, IFNAR1, TNFRSF1B, CCL17, IFNL1, IL16, IL1RL1, ILK, CCL25, ILDR2, CXCR1, IL36RN, IL34, TGFB1, IFNG, IL19, ILKAP, BMP2K, CCR10, ILDR1, EPO, CCR7, IL17C, IL23A, CCR5, IL7, EPOR, CCL13, IL2RG, IL31RA, TNFAIP6, IFNL2, BMP1, IL12RB1, TNFAIP8, IL4R, TNFRSF6B, TNFAIP8L1, TNFRSF10B, IFNL3, CCL5, CXCL6, CXCL1, CCR3, TNFSF11, CSF1R, IL21, IL1RAP, IL12RB2, CCL1, IL17RA, CCR1, IL1RN, TNFRSF11B, TNFRSF14, IL13, IL2RB, BMP8B, CCL2, IL24, IL18RAP, TGFBI, TNFSF10, TNFRSF11A, CXCL5, IL5RA, TNFSF9, IL1RL2, TNFRSF13C, IL36G, IL15RA, TNFRSF21, CXCL8, IL22RA2, TNFAIP8L2, IL18R1, IFNLR1, CXCR6, CCL3L3, TNFRSF1A, IL17RE, IFNGR2, IL17RC, TNFAIP8L3, ILVBL, TGFBRAP1, CCL4L1, CSF2RA, CCRN4L, CCL26, TNFAIP1, CCRL2, IFNA10, TNFRSF17, IFNA13, IL20, IL18BP, CCL3L1, TNFSF12-TNFSF13, IL5, IL23R, IL26, TNF, TGFA, CSF2, IL1F10, CXCL17, TNFSF13, IFNA4, IL37, IL12A, IL7R, IFNA1, IL1A, IL4, IL2, CCL22, CSF3R, IL10, IFNK, TGFB2, IL1R2, IL1B, IL17F, IL27RA, IL15, TNFSF8, IL36B, XCL1, CXCL16, TNFRSF19, IL3, CCL3, IFNA2, BMPR1B, IFNA21, TNFSF18, CCL8, IL17RB, TNFRSF25, IL22, IL10RB, IFNAR2, CCL18, IFNA16, CSF2RB, IL36A, TNFAIP3, IL13RA2, IL13RA1, CCR9, TNFRSF10A, IFNA7, IFNW1, XCL2, TNFSF14, CCR2, BMP15, BMP10, CCL15-CCL14, TGFBR1, IFNA5, BMP7, IFNA14, IL20RB, IL10RA, IFNA17, CCR6, TGFB3, CCL15, CCL4, CCL27, TNFRSF13B, TNFAIP2, IL31, IL17A, TNFSF15, CCL19, IFNA6, IL9 |
|  | CD8+ T cells | CD8A |
|  | Check-point | IDO1, LAG3, CTLA4, TNFRSF9, ICOS, CD80, PDCD1LG2, TIGIT, CD70, TNFSF9, ICOSLG, KIR3DL1, CD86, PDCD1, LAIR1, TNFRSF8, TNFSF15, TNFRSF14, IDO2, CD276, CD40, TNFRSF4, TNFSF14, HHLA2, CD244, CD274, HAVCR2, CD27, BTLA, LGALS9, TMIGD2, CD28, CD48, TNFRSF25, CD40LG, ADORA2A, VTCN1, CD160, CD44, TNFSF18, TNFRSF18, BTNL2, C10orf54, CD200R1, TNFSF4, CD200, NRP1 |
|  | Cytolytic activity | PRF1, GZMA |
|  | DCs | CCL17, CCL22, CD209, CCL13 |
|  | HLA | HLA-E, HLA-DPB2, HLA-C, HLA-J, HLA-DQB1, HLA-DQB2, HLA-DQA2, HLA-DQA1, HLA-A, HLA-DMA, HLA-DOB, HLA-DRB1, HLA-H, HLA-B, HLA-DRB5, HLA-DOA, HLA-DPB1, HLA-DRA, HLA-DRB6, HLA-L, HLA-F, HLA-G, HLA-DMB, HLA-DPA1 |
|  | iDCs | CD1A, CD1E |
|  | Inflammation-promoting | CCL5, CD19, CD8B, CXCL10, CXCL13, CXCL9, GNLY, GZMB, IFNG, IL12A, IL12B, IRF1, PRF1, STAT1, TBX21 |
|  | Macrophages | C11orf45, CD68, CLEC5A, CYBB, FUCA1, GPNMB, HS3ST2, LGMN, MMP9, TM4SF19 |
|  | Mast cells | CMA1, MS4A2, TPSAB1 |
|  | MHC class I | B2M, HLA-A, TAP1 |
|  | Neutrophils | EVI2B, HSD17B11, KDM6B, MEGF9, MNDA, NLRP12, PADI4, SELL, TRANK1, VNN3 |
|  | NK cells | KLRC1, KLRF1 |
|  | Parainflammation | CXCL10, PLAT, CCND1, LGMN, PLAUR, AIM2, MMP7, ICAM1, MX2, CXCL9, ANXA1, TLR2, PLA2G2D, ITGA2, MX1, HMOX1, CD276, TIRAP, IL33, PTGES, TNFRSF12A, SCARB1, CD14, BLNK, IFIT3, RETNLB, IFIT2, ISG15, OAS2, REL, OAS3, CD44, PPARG, BST2, OAS1, NOX1, PLA2G2A, IFIT1, IFITM3, IL1RN |
|  | pDCs | CLEC4C, CXCR3, GZMB, IL3RA, IRF7, IRF8, LILRA4, PHEX, PLD4, PTCRA |
|  | T cell co-inhibition | BTLA, C10orf54, CD160, CD244, CD274, CTLA4, HAVCR2, LAG3, LAIR1, TIGIT |
|  | T cell co-stimulation | CD2, CD226, CD27, CD28, CD40LG, ICOS, SLAMF1, TNFRSF18, TNFRSF25, TNFRSF4, TNFRSF8, TNFRSF9, TNFSF14 |
|  | T helper cells | CD4 |
|  | Tfh | PDCD1, CXCL13, CXCR5 |
|  | Th1 cells | IFNG, TBX21, CTLA4, STAT4, CD38, IL12RB2, LTA, CSF2 |
|  | Th2 cells | PMCH, LAIR2, SMAD2, CXCR6, GATA3, IL26 |
|  | TIL | ITM2C, CD38, THEMIS2, GLYR1, ICOS, F5, TIGIT, KLRD1, IRF4, PRKCQ, FCRL5, SIRPG, LPXN, IL2RG, CCL5, LCK, TRAF3IP3, CD86, MAL, LILRB1, DOK2, CD6, PAG1, LAX1, PLEK, PIK3CD, SLAMF1, XCL1, GPR171, XCL2, TBX21, CD2, CD53, KLHL6, SLAMF6, CD40, SIT1, TNFRSF4, CD79A, CD247, LCP2, CD3D, CD27, SH2D1A, FYB, ARHGAP30, ACAP1, CST7, CD3G, IL2RB, CD3E, FCRL3, CORO1A, ITK, TCL1A, CYBB, CSF2RB, IKZF1, NCF4, DOCK2, CCR2, PTPRC, PLAC8, NCKAP1L, IL7R, 6-Sep, CD28, STAT4, CD8A, LY9, CD48, HCST, PTPRCAP, SASH3, ARHGAP25, LAT, TRAT1, IL10RA, PAX5, CCR7, DOCK11, PARVG, SPNS1, CD52, HCLS1, ARHGAP9, GIMAP6, PRKCB, MS4A1, GPR18, TBC1D10C, GVINP1, P2RY8, EVI2B, VAMP5, KLRK1, SELL, MPEG1, MS4A6A, ARHGAP15, MFNG, GZMK, SELPLG, TARP, GIMAP7, FAM65B, INPP5D, ITGA4, MZB1, GPSM3, STK10, CLEC2D, IL16, NLRC3, GIMAP5, GIMAP4, IFFO1, CFH, PVRIG, CFHR1 |
|  | Treg | IL12RB2, TMPRSS6, CTSC, LAPTM4B, TFRC, RNF145, NETO2, ADAT2, CHST2, CTLA4, NFE2L3, LIMA1, IL1R2, ICOS, HSDL2, HTATIP2, FKBP1A, TIGIT, CCR8, LTA, SLC35F2, IL21R, AHCYL1, SOCS2, ETV7, BCL2L1, RRAGB, ACSL4, CHRNA6, BATF, LAX1, ADPRH, TNFRSF4, ANKRD10, CD274, CASP1, LY75, NPTN, SSTR3, GRSF1, CSF2RB, TMEM184C, NDFIP2, ZBTB38, ERI1, TRAF3, NAB1, HS3ST3B1, LAYN, JAK1, VDR, LEPROT, GCNT1, PTPRJ, IKZF2, CSF1, ENTPD1, TNFRSF18, METTL7A, KSR1, SSH1, CADM1, IL1R1, ACP5, CHST7, THADA, CD177, NFAT5, ZNF282, MAGEH1 |
|  | Type I IFN Response | DDX4, IFIT1, IFIT2, IFIT3, IRF7, ISG20, MX1, MX2, RSAD2, TNFSF10 |
|  | Type II IFN Response | GPR146, SELP, AHR |
|  | IFN gamma | IDO1, CXCL10, CXCL9, HLA-DRA, ISGF-3, IFNG |
|  | CTL | CD8A, CD8B, GZMA, GZMB, PRF1 |
| Cell functional signatures | Angiogenesis | ACVRL1, JAG1, ANGPT1, ANGPT2, CD34, CDC42, MAPK14, TYMP, EDN1, EFNB2, EGR3, EPHB4, PTK2B, FGF2, FGFR1, VEGFD, FOXC2, FLT1, FLT4, FN1, GPLD1, NR4A1, ID1, ITGA5, ITGAV, ITGB1, KDR, LOXL2, MMP14, NOTCH1, PDGFA, PDGFRB, PGF, PIK3CA, PTGS2, PTK2, ROBO1, SHC1, SRF, TAL1, TDGF1, TEK, VAV2, VEGFA, VEGFC, FGF18, NRP1, SEMA5A, RAMP2, CIB1, ESM1, JMJD6, HEY1, ADGRA2, GREM1, SRPX2, SOX18, PARVA, RNF213, E2F8, RSPO3, OTULIN, E2F7, CCBE1, BMPER, NRARP, TNFAIP6, VCAN, SPP1, CCND2, PIK3R1, STC1, JAG2 |
|  | Apoptosis | APAF1, ATF4, ATM, BAK1, BID, BOK, BRCA2, CASP2, CASP4, CASP9, DAPK3, DDIT3, E2F2, EP300, GSN, IFI16, INHBA, KRT8, LGALS9, NFATC4, P2RX7, PMAIP1, POLB, PPP2R1B, PPP2R5C, SORT1, SKIL, STK3, STK4, TIMP3, TNFRSF1A, TNFRSF1B, TP53BP2, DYRK2, CRADD, BCL10, AIFM1, BCL2L11, DNM1L, BCAP31, SIVA1, MLLT11, RIPK3, SNW1, PPP1R13B, PHLDA3, NUPR1, BBC3, CIDEB, TNFRSF21, PYCARD, CDIP1, G0S2, SHISA5, TNFRSF12A, WWOX, DDIT4, USP28, MOAP1, AEN, TMEM109, APOPT1, BMF, DAB2IP, DEDD2, ST20 |
|  | Cell Cycle | ANLN, ANP32E, ATAD2, AURKA, BRIP1, CASP8AP2, CBX5, CCNA2, CCNB2, CCND1, CCNE1, CCNE2, CDC25C, CDC45, CDC6, CDCA2, CDCA3, CDCA8, CENPA, CENPE, CHAF1B, CKAP2L, CKAP5, CKS1B, CKS2, CLSPN, DHFR, DLGAP5, DSCC1, DTL, ECT2, EXO1, FEN1, GAS2L3, GINS2, GTSE1, HELLS, HJURP, HMMR, KIF11, KIF23, KIF2C, LBR, MCM4, MCM5, MCM6, MKI67, NASP, NCAPD2, NDC80, NEK2, NUF2, NUSAP1, PCNA, POLA1, POLD3, PRIM1, PSRC1, PTTG1, RAD21, RAD51AP1, RANGAP1, RFC2, RFC4, RPA2, RRM1, RRM2, SLBP, SMC4, TACC3, TIPIN, TMPO, TOP2A, TTK, TUBB4B, TYMS, UBR7, UHRF1, UNG, USP1, WDR76, POLA2, GCLM, HMGB3, DEPDC1B, PRR11, NUCKS1, NUP37, UBE2T, ORC6, CENPM, VRK1, CDC25B, FAM83D, ASF1B, GRPEL1, RFC5, SRSF3, MND1, ZWINT, TUBG1, ENOSF1, RAN, RFC3, E2F5, AKIRIN2, TROAP, BORA, PIF1, FANCI, LYAR, CDCA5, DSN1, CCNF, CDC25A, SAP30, CDCA7L, INTS8, MCM7, CHAF1A, PBK, DTYMK, APEX2, KIF5B, SHCBP1, ZWILCH, KPNA2, H2AFX, H1F0, TUBB, HIST1H4C, PRC1, CENPW, MRPS18B, MZT1, KIFC1, KIF22 |
|  | Differentiation | ABL1, ACVR1, ADA, AGT, AKT1, ANXA1, ATOH1, ATP2B2, ATP7A, AXL, BCL2, BCL3, PRDM1, BMP2, BMP6, BRAF, CASP8, CAV3, RUNX2, RUNX1, CCNB1, CD86, CDK1, CDC42, CDH2, CEBPA, CEBPB, CREB1, MAPK14, CSF1, CSF1R, CSF2, DRD1, EDN3, EDNRB, EPHA2, EMX1, EP300, ERBB4, ERCC2, FGF8, FGFR1, FGFR2, FOXC2, FLT3LG, MTOR, GATA1, GATA4, GATA6, GDNF, GNAS, HDAC2, NCKAP1L, NRG1, HHEX, HIF1A, HMGB1, FOXA1, HOXA5, IGF1, IL2, IL4R, IL11, IL12B, IL15, ILK, INSM1, IRF4, JAG2, JUN, JUNB, KIT, LAMB2, LEP, LGALS1, LGALS9, LHX1, LIF, LMNA, LYN, SMAD4, MEF2A, MEF2C, KITLG, MMP14, MYF5, MYF6, MYH9, MYO7A, MYOD1, MYOG, NFATC2, NFIB, NTRK1, NTRK2, PAFAH1B1, PAX2, PF4, PITX2, PLS3, POU4F3, PPARG, MAPK3, MAP2K1, PROX1, PSEN1, PTGER4, PTN, PTPN11, PTPRZ1, RAC1, RAG1, RB1, RELB, RXRA, CCL19, SHOX2, SNAI1, SOD1, SOX4, SOX10, SOX11, SP3, SPI1, SRF, STAT5B, STK11, SYK, TAL1, TGFB2, TGFBR2, THRA, NKX2-1, TRAF6, TSC1, VEGFA, WNT7A, WNT8A, WNT11, WT1, ZAP70, EOMES, SEMA7A, TNFSF11, TP63, TNFSF9, FADD, NRP1, PROM1, SLC9A3R1, ATG5, SLC4A7, MAML1, ZEB2, TSPAN2, SEC24B, BATF, ZBTB1, KDM1A, KDM6B, HEY2, SCRIB, TENM4, BLOC1S6, FOXP1, BMP10, IL20, CDON, WDPCP, LEF1, IL23A, CLIC5, DLL4, TMEM100, MCOLN3, SOX6, MESP1, CYP26B1, FAM20C, OVOL2, SAV1, SEMA4A, BCL11B, NKAP, PDZD7, WNT5B, DCSTAMP, GDPD5, SLITRK6, MYLK3, TMC1, OCSTAMP, IL31RA, IL23R, GDF7, NKX2-3, STRC, ARX, GSX2, LHFPL5, ATP11C |
|  | DNA damage | BRCA1, MRE11, FOXN3, HIPK2, SP100, PSME4, RPS6KA6, TP63, GTSE1, PPP2R5C, TP73, CNOT4, DNAJA1, BAX, UIMC1, AURKA, CNOT3, PCBP4, TFAP4, E2F1, RGCC, RBL2, NBN, GML, BABAM1, CASP2, CDK5RAP3, CCND1, FOXM1, CDKN1B, CNOT2, ING4, CNOT6, TFDP2, NEK11, PSMD14, ARID3A, MAD2L2, GADD45A, SYF2, RPA2, PLAGL1, RBBP6, CDK2, CDKN1A, SOX4, CNOT1, PRMT1, BCL2L2, PCNA, RBM38, CCNB1, TAOK3, MDM2, CNOT6L, BRCA2, PML, FANCI, TP53, PMAIP1, CARM1, DTL, RPS27A, CNOT9, CCNA2, PLK2, TNKS1BP1, RPS3, ATM, CHEK1, HMGA2, UBC, CENPJ, XPC, CNOT8, BABAM2, CDC25C, CNOT11, BTG2, ZNF385A, USP1, ABRAXAS1, RNF168, SPIDR, E2F7, CRADD, CDK1, UBB, TRIAP1, RAD9A, KAT5, PLK3, ATR, SFN, HIC1, PIDD1, UBE2N, NPM1, CNOT10, CHEK2, FOXO4, MUC1, BRCC3, BLM, TFDP1, MDM4, CNOT7, E2F4, UBA52, PRKDC |
|  | DNA repair | RAD52, POLR2J, LIG3, REV3L, BRCA1, ERCC1, MNAT1, MRE11, RFC1, TDP1, RRM2B, ERCC8, RFC2, POLD1, NTHL1, TP53BP1, POLB, XRCC1, XAB2, POLD3, XRCC5, RIF1, PALB2, SIRT1, POLR2E, POLR2F, POLE2, APEX1, POLI, POLA1, POLR2C, MPG, NBN, ERCC2, POLR2I, LIG1, RPA3, POLD2, RAD51C, GTF2H1, RAD51AP1, GTF2H3, RFC5, FANCE, RAD50, OGG1, FANCL, MAD2L2, RPA2, RAD23B, POLK, SMUG1, WRNIP1, XRCC3, MBD4, POLN, RPA1, PCNA, MUTYH, RFC3, CDK7, CCNH, DDB2, REV1, BRIP1, XPA, TDG, BRCA2, TP53, PARP1, RPS27A, POLR2D, FANCD2, CETN2, POLR2K, SSRP1, ATM, XRCC4, CCNO, XPC, FANCC, ERCC3, POLR2H, RFC4, HMGB2, WRN, POLL, ALKBH3, DDB1, POLR2G, FEN1, UBE2V2, MGMT, POLH, EXO1, LIG4, ATR, POLD4, ERCC4, POLE, TOP3A, UBE2N, FANCB, RAD51B, FANCF, RAD51D, FANCA, FANCM, H2AFX, ALKBH2, HMGB1, XRCC6, XRCC2, BLM, GTF2H4, FANCG, UBA52, PRKDC, GTF2H5 |
|  | EMT | BGN, CDH11, CDH2, COL1A2, COL3A1, COL5A2, CTGF, DDR2, ECM1, FAP, FBLN5, FBN1, FOXC2, FSTL1, GSC, HAS2, IGFBP4, MMP1, MMP2, MYL9, NR2F1, PCOLCE, POSTN, PRRX1, PTX3, RGS4, SNAI1, SPOCK1, TAGLN, TUBA1A, TWIST1, VIM, WNT5A, ACTA2, BRCA1, SERPINH1, CENPA, COL1A1, COL4A1, COL6A2, VCAN, CTSB, DAB2, DCK, FBLN2, FN1, GAS1, HIF1A, HMMR, TNC, ITGAV, LUM, MCM3, MCM7, MKI67, MMP14, NT5E, ODC1, PDGFRA, PLAUR, PPIC, HTRA1, RBL1, CXCL6, SDC1, SDCBP, TIMP1, ENC1, TUBB3, PLK4, ORC6, ANLN, CAMK2N1, ENOPH1, CD44, LOXL2, TGFBR3, WNT16, WNT2, TGFBR1, FGF8, EPB41L5, OLFM1, HEY2, LEF1, ERG, HEY1, TMEM100, EFNA1, S100A4 |
|  | Hypoxia | PLIN2, ADM, ADORA2B, AK4, ALDOC, ANG, ATF3, BNIP3, BNIP3L, CA9, CA12, CAV1, CCNG2, KLF6, DUSP1, EFNA1, ENO2, F3, FOS, NR3C1, GYS1, HIF1A, HK1, HK2, HMOX1, IGFBP3, IL6, JUN, LDHA, LOX, MIF, MXI1, NFIL3, P4HA1, SERPINE1, PAM, PDGFB, PDK1, PFKFB3, PFKFB4, PFKP, PGF, PGK1, PGM1, PLAUR, PLOD2, PPP1R3C, RNASE4, SAT1, SLC2A1, SLC2A3, SPAG4, STC1, TGFBI, TPI1, VEGFA, VLDLR, CXCR4, PPFIA4, BHLHE40, KLF7, STC2, SAP30, P4HA2, STBD1, AKAP12, CITED2, NDRG1, TXNIP, ZNF292, JMJD6, MAFF, FAM162A, HILPDA, ERO1A, ANGPTL4, DDIT4, EGLN1, KLHL24, TMEM45A, KDM3A, ZNF395, EGLN3 |
|  | Inflammation | BMP2, CD6, CEBPB, CCR7, CSF1, F3, F12, FCER1G, B4GALT1, FFAR2, CXCL1, CXCL2, CXCL3, ICAM1, IL1A, IL1B, IL6, CXCL8, IL12B, TNFRSF9, IL18, CXCL10, IRAK2, KLKB1, NFKB1, NFKB2, OLR1, OSM, SERPINE1, MAP2K3, PTGS2, PTX3, REL, RELA, RELB, S100A8, CCL2, CCL3, CCL4, CCL5, CCL7, CCL8, CCL18, CCL20, CCL23, CXCL6, CXCL11, THBS1, TLR2, TNF, TNFSF4, VCAM1, WNT5A, RIPK2, SPHK1, CCRL2, OSMR, RASGRP1, TNIP1, KDM6B, TLR7, IL23A, TNIP2, ZC3H12A, CCR2, ACOD1, JAG1, ATP2B1, CD80, CD44, CSF3, CD55, EDN1, EREG, ACSL1, FUT4, GCH1, GPC3, IL1R1, ITGB8, LIF, MARCKS, NFKBIA, NINJ1, SERPINB2, PLAUR, RGS16, SDC4, SLC1A2, FSCN1, STAT5A, TNFAIP2, BTG2, FOSL1, SNN, PLPP3, TNFSF9, IER3, SLC28A2, TRIP10, LITAF, NAMPT, KIF1B, DENND5A, ICOSLG, PPP1R15A, MAFF, HIPK2, G0S2, DRAM1, TSLP, RNF144B |
|  | Invasion | AEBP1, AKR1B1, AMD1, SLC25A5, ATP5PB, BAG1, BGN, C1QB, CALD1, CAPG, CCNE1, CDH11, CKS1B, CKS2, COL1A1, COL1A2, COL3A1, COL5A1, COL5A2, COL6A2, COL6A3, COL10A1, COL11A1, COMP, CSE1L, VCAN, CTSK, DAB2, DDX5, EDNRA, FAP, FBN1, FN1, GNAS, H2AFZ, HMGB2, HNRNPU, HSD17B4, CYR61, INHBA, LAMB1, LAMC1, LGALS1, LOX, LOXL2, LUM, MMP2, MMP11, HNRNPM, NDUFB7, YBX1, PDGFRB, PLAU, PRRX1, PNN, PPIC, PROS1, PSMA2, PSMB4, RGS4, SNAI2, SPOCK1, TGFBI, THBS2, THY1, TNFAIP6, UBE2V2, ADAM12, MFAP5, ITGBL1, TP53I3, NUAK1, HNRNPDL, TXNDC9, LRRC17, IFI30, POSTN, CBX1, NID2, RRAS2, RALY, SEPHS2, HEY1, MXRA5, OLFML2B, TMEM158, WWTR1, GREM1, NOX4, CLEC4A, COPZ2, ASPN, CEMIP, CRISPLD2, TUBB6, LRRC15, TUBB |
|  | Metastasis | ACKR3, AFP, AGR2, AKT1, ALDH1A1, ANGPTL4, ANXA1, ANXA2, AQP5, ATF3, AURKA, AXL, B2M, BMI1, BMP2, BRAF, BSG, CA9, CCDC88A, CCND1, CCR7, CD24, CD274, CD44, CDCP1, CDH17, CDH2, CEACAM1, CEACAM5, CLIC1, CRP, CTGF, CTNND1, CTSB, CTSK, CTSL, CTTN, CXCL12, CXCL2, CXCL8, CXCR2, CYR61, DDR2, EGF, EGFR, EGR1, EPAS1, EPCAM, ERBB2, ERBB3, ESR1, ESR2, EZH2, EZR, F2RL1, FGFR1, FLOT2, FLT1, FLT4, FN1, FOXC2, FOXM1, GLI1, HGF, HIF1A, HMGA1, HMGA2, HPSE, ID1, IDO1, IGF1R, IGF2BP3, IL6, ITGA3, ITGA5, ITGA6, ITGAV, ITGB1, ITGB3, JAG1, KDR, KRAS, KRT19, L1CAM, LAMC2, LASP1, LGALS3, LOX, LOXL2, MACC1, MCAM, MDM2, MET, MIF, MKI67, MME, MMP1, MMP11, MMP13, MMP14, MMP2, MMP3, MMP7, MMP9, MSN, MST1R, MTA1, MTDH, MUC1, MYC, NEDD9, NOTCH1, NTRK2, PCNA, PDGFRA, PDPN, PIK3CA, PKM, PLAU, PLAUR, POSTN, PRKCI, PROM1, PSCA, PTGS2, PTHLH, PTK2, PTP4A3, PTTG1, PXN, RAB25, RAC1, RELA, RHOC, ROCK1, S100A4, SATB1, SDCBP, SELE, SELP, SLC2A1, SNAI1, SNAI2, SNCG, SOX4, SPARC, SPP1, SRC, STAT3, STMN1, TERT, TGFBI, TGM2, TIMP1, TMPRSS4, TNFSF11, TYMP, VCAN, VEGFA, VEGFC, VEGFD, VIM, WASF3, YAP1, ZEB1, ZEB2 |
|  | Proliferation | ADK, BAX, CCND1, BMPR1A, BUB1B, CCNA2, CCNB1, CD40, CDK1, CDC20, CYBA, HMGB2, IGFBP2, MKI67, PRKD1, TEK, TK1, TOP2A, DLGAP5, MELK, GINS1, KIF14, RACGAP1, ASPM, CD24, CX3CL1, IGF1, CD74, TP63, FGFR1, DCT, PTPRC, LHX5, ESR1, ABL1, XBP1, PDGFB, HIF1A, CD40LG, FGF9, FZD3, RIPK2, NUMBL, EBI3, WNT2, IL2, IL23A, VEGFA, SEMA5A, IL4, CD86, STAT1, ID2, DOCK7, SLAMF1, TNFSF4, WNT1, TNFSF9, MED1, SMO, TLR4, MYC, LEF1, ASCL1, FGF7, OSR1, IL18, WNT3A, WNT7A, DISC1, BMP10, SYK, IL13, BCL2, ID4, STAT5B, CD34, CTC1, TBX1, IRS2, TNFRSF4, HMGB1, ZNF335, NRARP, BMPR2, AGER, MIF, CCL5 |
|  | Quiescence | ALOX5, ANXA5, AREG, CREM, CRHBP, EMP1, EVI2A, GBP2, CXCL1, CXCL2, CXCL3, GUCY1A1, H1F0, HBB, HLX, IL1B, CXCL8, INHBA, CXCL10, JUN, TM4SF1, MLLT3, NR4A2, SERPINB2, PCDH9, PMCH, PTGS2, PTPRC, RGS1, RHAG, TSPAN31, CCL8, CCL19, CCL20, CXCL6, CXCL11, CXCL5, TRA2B, SPTBN1, SVIL, TCF7L2, TFPI, TGFB1I1, TPBG, TNFSF4, FXR1, NRIP1, HIST1H2AC, HIST2H2BE, PPFIBP1, TNFSF10, IL18R1, VNN1, CYTIP, OPTN, MPZL2, CXCL13, NFAT5, GLIPR1, RBPMS, FNBP1, MAFF, TRA2A, TPSB2, ZBTB10, HIST1H2BK |
|  | Stemness | AFMID, AFP, ANPEP, APC, AQP1, ASCL1, ASCL2, AXIN2, AZGP1, BAZ2B, BMI1, BOC, C6orf62, CA2, CAMK2N1, CCL5, CCND2, CD200, CD24, CD33, CD38, CD44, CDCA7, CDK6, CEBPA, CFTR, CHD7, CORO1C, CXCL2, DBX1, DBX2, DNMT3A, DPP4, EBF1, EEF1A1, EGFR, EIF4B, EMX1, EMX2, ENG, EPHB2, ETS2, ETV1, EVI2A, EZH2, FABP7, FAM84A, FBLIM1, FBXO27, FERMT1, FOXA2, FOXA3, FOXG1, GATA1, GATA2, GATA3, GATA4, GFAP, GFI1, GLI1, GPC3, GPM6A, GPSM2, GPX2, H19, H3F3B, HACD3, HAPLN1, HEPN1, HES1, HNRNPH1, HNRNPL, HOPX, ICAM1, IDH1, IKZF1, IRX3, ITGAM, KDR, KIT, KLK10, KRT14, LATS2, LGR5, LOH12CR2, LRIG1, LY6D, LYZ, MALAT1, MBOAT1, MESP1, MESP2, METTL3, MLLT10, MME, MYB, MYC, NANOG, NCAM1, NEK5, NELL2, NFE2, NFIA, NFIB, NKX2-5, NODAL, NT5E, OLFM4, OPHN1, ORC6, PABPC1, PAX6, POU5F1, PROM1, PTK7, PTMA, PTPRC, PTPRG, PTPRO, PTPRS, PTPRZ1, QPCTL, RAB42, RAMP2-AS1, RBM6, RGMB, RNF43, RUNX1, SET, SLC12A2, SMAD2, SMOC2, SOX1, SOX11, SOX17, SOX2, SOX3, SOX4, SOX9, SPDYE1, SPDYE5, SPHKAP, SRGAP2C, STMN1, STMN2, TAL1, TATDN3, TCF12, TCF4, TDGF1, TFDP2, TFRC, THY1, TNFAIP8L1, TOX3, TRA2A, TSPAN6, UGT8, VEGFA, ZBTB8A, ZNF793, ABCG2, ALDH1A1, ALDH1A3, DNER, MET |

**Abbreviations:** PA, pituitary adenoma; aDCs, activated dendritic cells; APC, antigen presenting cell; CCR, chemokine receptor; HLA, human leukocyte antigen; iDCs, immature dendritic cells; MHC, major histocompatibility complex; NK cells, natural killer cells; pDCs, plasma dendritic cells; Tfh cells, follicular T-helper cells; Th1 cells, T-helper 1 cells; Th2 cells, T-helper 2 cells; TIL, tumor infiltrating lymphocyte; Treg, regulatory T cells; IFN, Interferon; CTL, cytotoxic T lymphocyte.
