# Supplementary figures and images for: Multi-Omics Investigations Revealed Underlying Molecular Mechanisms Associated With Tumor Stiffness and Identified Sunitinib as a Potential Therapy for Reducing Stiffness in Pituitary Adenomas
Source: Front Cell Dev Biol. 2022 Mar 15;10:820562. doi: 10.3389/fcell.2022.820562 (PMC8965615; doi:10.3389/fcell.2022.820562)

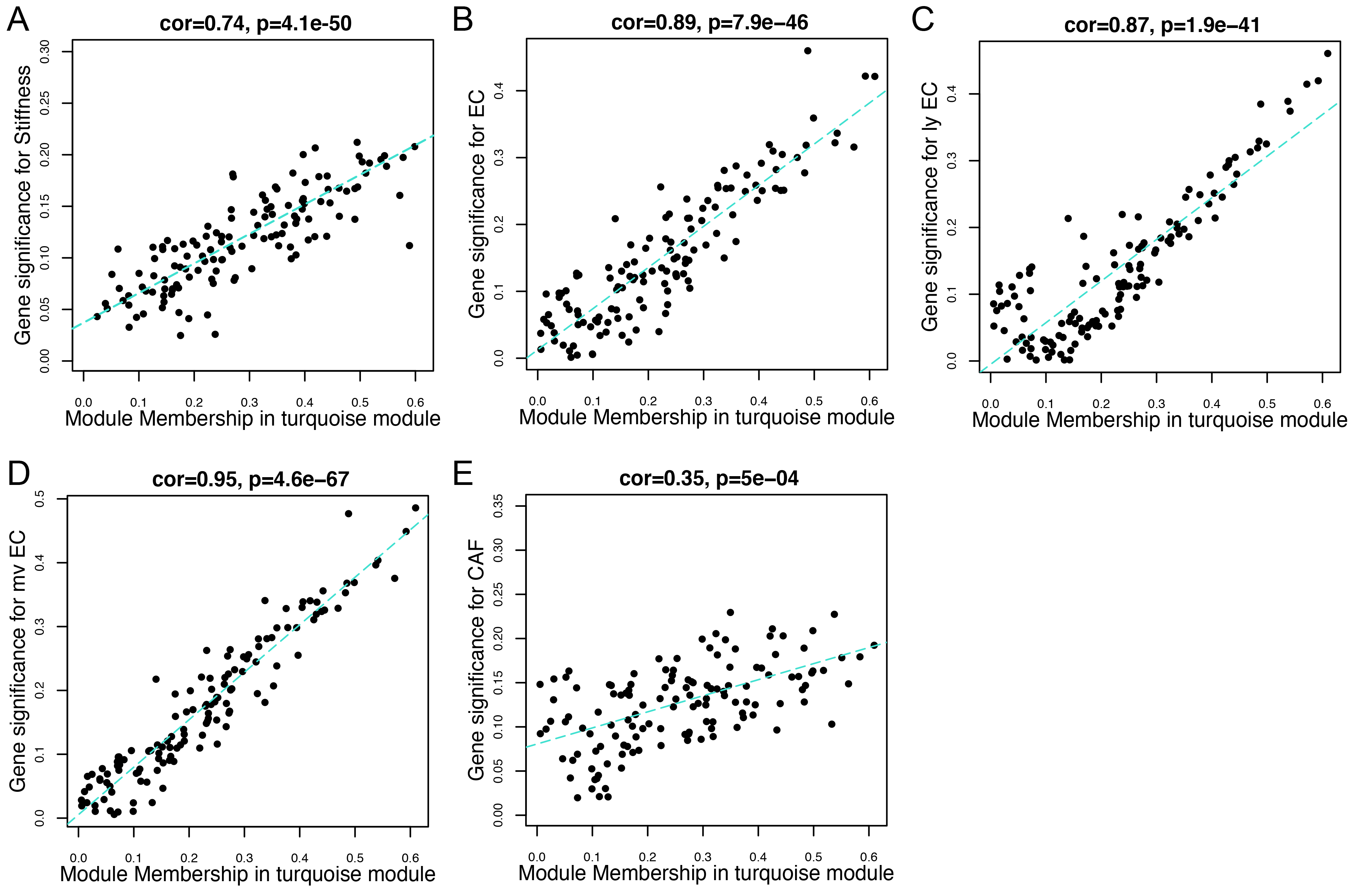

Supplement: Supplementary file 2 [file Image2.TIF]

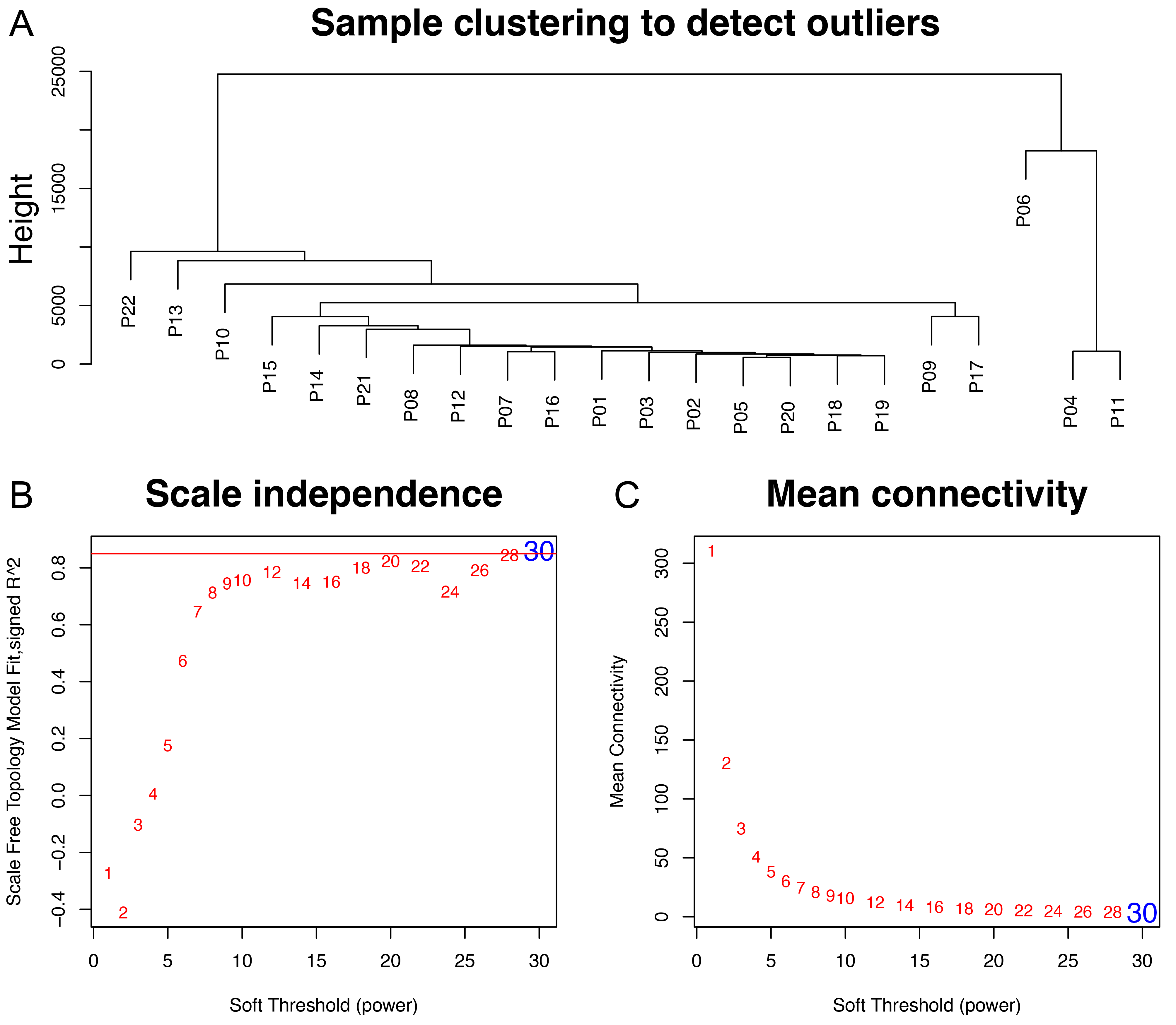

Supplement: Supplementary file 3 [file Image1.TIF]
