# Supplementary material for: Multi-Omics Investigations Revealed Underlying Molecular Mechanisms Associated With Tumor Stiffness and Identified Sunitinib as a Potential Therapy for Reducing Stiffness in Pituitary Adenomas
Source: Front Cell Dev Biol. 2022 Mar 15;10:820562. doi: 10.3389/fcell.2022.820562 (PMC8965615; doi:10.3389/fcell.2022.820562)
Supplement: Supplementary file 4 [file Table2.DOCX]

**Supplementary Table 2.** The epigenes of each module.

| Module | Gene symbols |
| --- | --- |
| Turquoise (SRGs) | BGN, RARRES2, IGFBP4, MYL9, TPM2, MGP, COX4I2, CA4, SLPI, ADIRF, GGT5, ESAM, ICAM2, NUPR1, CAVIN3, IFITM2, TMEM204, LAMB2, VWA1, SELENBP1, HP, FAM167B, ENG, SMIM24, EMILIN1, PLVAP, NES, BPIFB4, EVA1B, CDC42EP5, EFNA1, PPP1R14A, ECSCR, LCN2, INMT, TRIP6, CALY, S100A4, COMTD1, TMEM88, APLNR, SERGEF, AL035071.1, COLEC11, ANGPTL4, DNASE1L3, HTRA3, AC073896.4, CORO7, SIPA1, ASPDH, CLDN5, ZNF358, NDUFA11, KANK3, LIMS2, REM1, PCOLCE, ROBO4, TCIRG1, GMFG, HES1, SCGB3A1, AC006942.1, SOX18, GRASP, VAMP5, CDC42EP1, TNFRSF4, RSPH9, HLA-DRB1, RAMP2, RPL21P16, FAM212A, PLPP2, FXYD5, COX7A1, FLT4, H2AFJ, DPM3, CLEC11A, FTH1P8, TMEM190, CCDC85B, EFCC1, AP001269.4, CRYAB, EXOC3L1, PYCARD, RBP5, CHADL, C5orf66-AS1, RASIP1, TMEM225B, MT3, LY6H, TMEM91, S100B, FUOM, DUSP15, CERS1, GSG1L, C4orf48, LINC00634, PRR22, CRYGD, RPS7P10, RAMP1, SPON2, FAM183A, RPL5P34, RRAD, UBXN10-AS1, AC108673.3, SCARNA7, AC018738.1, GRAP, SLC39A4, FNDC10, LINC01863, AL355075.4, AL731533.2, TMPRSS9, CHI3L1, PNMT, RNU1-4, TRBV30, AC105446.1, RNU1-2, S100A1, SAA1 |
| Salmon | COL12A1, PRL, PDGFRB, BAG1, TMEM150C, NBL1, RIPOR3, FOS, ZP1, GAS6, NOTCH3, COX6A2, CGREF1, CST6, SSTR5, FAM163A, AC131097.3, TMEM243, SELENOS, C1QTNF1, COL4A3, PHYHIP, IGFBP7, RASA3, PCSK6, ANKRD9, PLXDC1, FOXQ1, TRIM7, KCND2, C11orf24, AC012368.1, SLC1A5, C10orf10, COL4A4, KCNQ1, LHFPL6, TBX2, AC099681.1, GFRA3, GALNT17, SPINK4, CD99, KCNJ8, NEU4, CGNL1, COL3A1, ECM1, ARG2, COL9A2, KCTD15, SPRY4-AS1, LINC01315, RRBP1, PDGFRL, VDR, FAM162B, GEM, PYCR1, SPRY4, TOX2, AL133346.1, KRT42P, AC020916.1, MRVI1, PCDHGB6, CRTAC1, KCNK12, DAAM2, FJX1, MFSD2A, RET, NOV, AL450124.1, SNHG18, MIR27B, GPR12, HCFC1-AS1, S1PR3, DLK1, MIR770, RTN4RL2, SAMD4A, SLC35F3, PCDHB8, ARHGAP36, CDKN2B, BVES, PLCXD3 |
| Red | GZMK, SSTR1, HRK, RASSF6, KCNE4, PRKCQ-AS1, JUN, SRC, ESM1, AL359182.1, CLDN4, PRKCQ, CDK5R1, TMEM72, PLCH2, U62317.5, AC005618.1, RAB3B, ZNF365, PLP1, PKIA, ANGPTL7, LINC01197, SMAD3, REN, AC084816.1, NCKAP5, CXCL13, NEUROD1, EDA, U62317.1, PRKD3, CAMK1D, HMGN2P15, PXDC1, CBFB, ZNF385B, TMEM100, GPT, AP000695.2, NOS1, HHATL, ATP13A3, RILP, CALD1, SMAD6, ID4, PDE6A, TNFAIP8L3, MKRN2OS, USH1C, AC093278.2, METTL7A, TMEM31 |
| Pink | LTF, RCN3, PMP22, MSN, PTPN5, RAPGEF3, DKK1, FAM53A, PCK1, ITM2C, FNDC9, EMP2, ALDH1A2, AC018742.1, DOCK11, KCNS3, TCEAL5, FHL2, HMCN2, ABCB4, PENK, LINC01849, RENBP, NRN1, PPP1R15A, NID1, ANKRD26P1, OCIAD2, CARMIL3, MEG9, AC009549.1, CD55, PAPLN, AL135999.1, CTHRC1, CBLN1, AC093726.1, THBD, AC099681.2, PHACTR3, PTX3, ADRB2, AC022167.1, MIR153-1, TUBA1C, RELB, AL645924.1, FAM109B, MIR7-3, PAX5, GPRC5B, MYH6, GOLIM4, AC007614.3, KIF17, LMO4, CES5A, KCNH8, YPEL4, HVCN1, FAM133A, CDR2, PRKCH, FBLN7, GAD1, TCEAL9, CLVS1, DPYS, IKBIP, TMEM266, BACE2, MIR410, RCAN1, AC010624.3, SNAPC1, AC022167.2, NRROS, P4HA3, CCNO, GRM8, JAM2, MYRIP, CNGB3, C2CD4A, TDRD9, MEG3, LINP1, MIR656, CA14, TRPC3, BDNF, RNU7-57P, AC127455.1, MIR135A2, CASQ2, NR4A2, NR4A1, HLA-G, INHBE, ARX |
| Lightcyan | EZH2, AC010980.2, ESPN, ECEL1, RAB15, CTSV, GPC5, ASCL1, RAB37, PCDHA10, COL4A5, ERICH5, RASGRP3, LIFR, AL133163.3, SLIT3, MATN2, DPP4, PLA2R1, SLC37A2, GLDC, GLB1L3 |
| Grey | RGCC, DSCAML1, HEYL, SERPINE2, ANK1, ACTA2, SORCS2, TGFB3, FAM118A, ANKRD24, BEX5, JPH1, FOXS1, NELL1, NRG1, LRRC32, RABAC1, MT-TD, TAGLN, MRC2, TCEAL6, KDM1B, SOD3, FMNL2, LAMC3, TTC24, TNFAIP8L1, CCDC61, SMTN, MAPK15, PLEKHO1, GDPD5, RELL2, GJA4, AC026304.1, DHRS3, CLEC3B, FCHSD1, CA10, COL18A1, CD14, DDAH1, AL512625.2, PPL, LRP4, CRIM1, TMCC3, MIR126, SYT5, NRGN, CRYGEP, GSN, NEDD9, NMRAL1, ISLR, GCLC, MPZL2, DLL3, PTP4A3, NPIPB15, PTPRU, LIMA1, PRICKLE3, ETHE1, DOCK6, BRD9P2, RGS5, ARNTL, NLK, UNC5A, SYNPO, MYLIP, SYNDIG1L, A4GALT, ALS2CL, CD248, AC245297.2, MTMR9LP, LRIG3, STXBP2, CD8B, CCDC88C, ABCA1, LAMB1, PGF, AC096677.2, DDR2, AC005306.1, EHD2, MISP3, AC010478.1, PHGDH, SELENOM, F10, ARID5A, AC107959.1, RASL12, AFAP1, RPL39L, FBLN1, MXRA8, MMP14, WFDC1, RERG, GPD2, IFITM3, KRT19, ARFGEF3, PDE6H, CSNK2A3, HABP4, FN1, GSDMD, EFR3B, ANXA11, NACC2, CCDC151, PTH1R, PRKCA, PLXNC1, ANKRD34C-AS1, RERGL, WDR62, VSIR, TIMP3, VASN, OLFML3, SLC17A8, PROCR, MFGE8, SMCR8, COL5A3, CFC1, MMP2, HIST2H2BE, BCL2L11, IGSF11, SULF1, SHF, LAPTM4B, DDX6, SOX13, MEIS2, VMO1, CACNA1H, BCL2, ZBTB18, TEAD2, GRIA3, LMO3, ST18, GNG11, FAM91A1, TLCD1, LPIN3, MIR590, ZDHHC11, PPARGC1B, SERINC5, SAMD14, C17orf107, SEMA6A, KLHL11, CORO1A, CYP4F32P, POMK, ASAP2, AC007637.1, SLC35E2, MYH11, AC074212.1, IGFBP2, AIF1L, H1FX-AS1, VN1R108P, MARCKSL1, MREG, CBARP, PSTPIP2, S100A9, MALAT1, ZFX, THY1, OSBPL10, S100A6, DUSP23, SHB, SH3PXD2A, IER3, BBS10, AC136632.1, HOPX, DPP6, AC139887.1, LPAR2, LRCH1, CRHR2, BCO1, TAGLN2, VWA5B1, SLC22A15, FMOD, RPS6KA2, RASEF, SLC8A1, RPS6KA5, GUCA1C, MID1, MIR641, ZDHHC22, LINC01273, HLA-A, CITED2, LMCD1, RND3, SLX4IP, COLCA1, ZBTB26, C11orf96, GPM6A, VSTM2L, PCSK2, AL121929.2, EGFLAM, KCNH6, ADGRG1, CNN1, AC005062.1, ALOX5, TSPAN6, HRC, AC083795.2, RTBDN, FAM196B, AC093702.1, FAM201A, OLFML2A, ZDHHC14, DMGDH, CRYBA1, CALML4, TMEM238, FOXF2, WSCD1, EFCAB12, TAGLN3, APOL3, MIR93, SLC19A2, DGKG, CYTOR, KIAA1211L, WWC1, HERC2P3, SPOCK3, DDIT4L, FOSL2, CELF2, PLD5, ZNF883, AL121845.1, MISP, RAB38, AC087258.1, TGFBR3, PRKG2, AC124312.3, ADAMTS10, PRPS2, NAT16, ELN, COL6A2, TPSP2, TMEM159, PCDHGA6, ADRA2A, SYDE1, ANKRD29, RN7SL4P, S100A8, AC010890.1, KCNG4, BAALC-AS2, AMH, CCR10, SMIM22, RPS20P14, LINC00654, RMST, VAV1, PLD4, AC073464.2, PTPRG, COLGALT2, SCN1A, PTPRC, ITGA4, RASL11A, C3, CRHR1, SNORD14A, CNTN4, AC005034.4, RHOJ, MASP1, FAM166B, PGGHG, MAT1A, LINC01977, ELFN1, GALNT10, ASIC4, ESR1, CCDC173, KL, VWC2L, HR, RNA5SP474, SEL1L3, FLRT3, C3orf58, AC103770.1, XYLT1, FAM126A, HIST2H2BD, QPRT, ZFP92, TMEM182, GRIK2, STRA6, HS3ST4, AL590560.2, ADCY7, C22orf42, GPX3, RPS2P32, AL590617.2, GALNT13, AC099791.2, BICDL2, PNPLA7, FCN3, ADORA1, GLIS2-AS1, EPS8L1, SASH1, AC053503.1, AC069185.1, CD164L2, LINC02381, TMEM119, C5orf38, TRBV7-3, RGL3, HLA-DRB5, PDGFD, CYP4X1, AL009174.1, CDH12, LHB, SLC38A11, CDKN3, TGFBR3L, KLHL35, STEAP1, PLEKHG2, RAB19, GGN, FGF1, F3, ADAM19, CHL1, AC116348.2, SHH, IGDCC4, ANO4, F8, LINC02082, C2orf50, RHBDL2, C16orf74, PKP3, PCDHGA4, TRIM17, ANKRD2, VGF, SH3RF3, CNGA3, AC233723.1, LCN12, TDRD10, C10orf82, STAC, XIST, EMILIN3, AC148477.1, TOX3, C14orf144, MT1G, PCDHB4, LINC01833, CHAD, RNU6-118P, SYNPR, GSTM1, RNU6-8, ROR2, AP000695.1, AC104024.2, CLCNKB, TRBV9, AL035425.1, HTR1A, VSNL1, SORCS3, SOX9, CA12, IGSF1, RESP18, FOLR1, PRSS8, CLEC4GP1, KLK11 |
| Greenyellow | RAMP3, METRNL, GHSR, GH1, AL451062.1, NEAT1, ANKRD66, AC126177.4, GCNT3, VWA3A, CISH, CEP128, FGF7, GAS6-AS1, COCH, LINC00880, GRM7-AS1, AC113383.1, CD79B, CTGF, KDM4A-AS1, OTOS, TMEM45A, PSMB8, SRPX2, DEFB119, MAGEH1, NDUFAF2, PDIA5, DNAJB6, TMEM233, ABCB1, RASGRP1, NLRC5, CHST1, TNFRSF10C, GDF15, LINC00881, DIO2, WNT5B, PRLR, TMEM158, RAB33A, GPR161, RGS6, DNAJC13, AC073842.1, CEL, DOK5, THSD1, PSMB8-AS1, CDCP1, NMB, ELL2, APOL4, RARA-AS1, PTGS1, CADM2, KLHL13, FAM198B, KDELR3, SOCS2-AS1, AC090617.4, MYL10, PPFIBP2, 11-Mar, EAF2, ARL4A, GPR3, NPTX2, MAP6D1, AKR1B10, B3GNT5, FAM20A, AC002070.1, AC017100.1, MIR7-3HG, SLC44A3-AS1, C3orf67, SLC8A3, UGT3A2, PTPRE, SLC44A3, EGR1, GHRHR, GNLY, AC090617.6, CD99P1, AC104184.1, MYH15, EDIL3, PIP5K1B, PCSK1, ASB4, PTPRZ1, FAM90A1, GZMA, IL18R1, LINC02489, POU1F1, AL117190.1, HEPACAM2 |
| Green | RBP4, G0S2, CARTPT, TMEM37, FFAR4, SYCE1, AC026333.1, ASMTL-AS1, MT-TW, PEBP4, AC022390.1, IDH1-AS1, DBI, FABP5P7, SPACA6, MIR7-2, IGHA2, TPSAB1, LEFTY2, SYCE1L, MTND1P23, GNMT, AC018638.6, PRSS50, TCN2, AL109659.2, AC120498.3, TPSB2, IGHA1, AC124657.1, AC007292.1, AC244197.2, AC024060.1, FAM159B, TNNC2, GDPD2, CTXN2, AC024940.1, SPINK2, CCER2, SCGB1B2P, MTX1P1, AP005205.2, MT-TT, IGLC3, AL158152.2, FAAHP1, COL16A1, NMRK2, C2orf80, CES3, KCNIP3, CRYGC, CRNDE |
| Cyan | AVPR1B, AC010247.2, FGFR3, MSGN1, NWD1, PRR15L, CCNJL, AC141928.1, ADRA2C, MYBPHL, KLHDC8A, TBX19, AC005828.4, LMO1, HOMER2, PRPH, KANK4, AC016044.1, KCNIP1, CTSLP4, ALPK2, NNAT, SSC5D, AC009414.2, CACNA1A, AC008808.2, SAPCD2, BLCAP, SOX3, LINGO1, SCN1B, PCP4L1, STUM, TMEM184A, DDIT4, RAB26, LINC02432, CFAP58-AS1, HSPB6, PSRC1, PRR15, LINC02106, ODF3L1, HIST3H2A, MT1X, POP1, CHST2, DMRTA2, KCND1, COL28A1, AL645608.1, CD82, HIST3H2BB, CALCB, SST, AGT, PKNOX2, SAMD11, MMP23B, RXRG, RASD1, NPW, CIB2, NXPH4, WNT9A, CHRM4, HIF3A, HES6, GAP43, MT1E, MBL1P, TMEM176A, CALCA, GFOD1-AS1, IGSF21 |
| Brown | LRRN1, SPP1, KIAA0040, IGFBPL1, IRF6, CCNG2, CALB1, AC090796.1, RP1L1, AR, AGMO, SDK2, PTH, SESN3, ABCA12, SLC39A8, FASN, MLF1, SERTM1, TMEM2, PRDM10, PFKFB2, AC099805.1, GAS2, CERKL, PLEKHM3, ADGRG2, FMO5, LINC01550, EYA1, ARRDC4, ATF2, FRMPD1, PRTFDC1, AC005726.2, AF130351.1, NFIL3, NRXN1, NIM1K, PAQR8, SCD, CASK, ZDHHC2, FKBP5, ATP8A1, GFOD1, PDLIM5, EBF1, VSTM4, GNA13, RBM20, ARMC4, ENTPD3, MKX, MAPK6, GPR68, KIAA1211, NR2F2, GRIK1, ISL1, XK, MCC, AL121721.1, COL11A1, C15orf48, AC005726.4, RPRM, PLSCR4, BMP6, SLC1A3, RORB, IL5RA, OPCML, GLUL, L3MBTL4, CHRDL1, OSBPL1A, RGS4, NEFM, KCNQ5, PDE5A, RHOU, TLN2, FSTL5, PCDH9, VAV3, RALYL |
| Blue | ELSPBP1, CXCL14, BTG2, CRH, STC1, ITGA6, EGFL6, AL161911.1, NHSL2, OASL, PAGE2, IGKV1-6, TAC1, MAN2A1, TNC, RAB9B, SEZ6, CPD, CCL19, SAMD9L, OAS3, DDX60, CBLN4, STAT1, ZNF185, KIF12, IL6ST, LAMP3, RSAD2, UHMK1, SLC26A2, DENND4C, MED13, CORO2A, LNPEP, AKAP11, PARP9, GPC3, RNF213, TAOK1, LDLRAD4, CFI, SYT1, ZDBF2, SYT14, CSRNP3, ZDHHC20, KIT, ZBED6, DENND4A, HERC6, CD2AP, UFL1, RALGPS2, SNX10, APOOL, ENPP4, KLF10, ATF3, MTX3, IFI27, MANEA, LTBP1, MMP9, ERAP2, NEFH, AGL, MX2, SELL, DDX58, NR4A3, PDZK1, ACVR1C, KLHL15, CNKSR2, CXCL10, TSHB, ELK4, IFIT2, IFIT3, WEE1, TTC39C, PKIB, HSP90AA2P, OAS2, HIST1H2BC, IFI16, TMEM45B, PTPRK, PDK4, HERC5, CNTFR, RTP4, CPNE8, F2RL1, RFPL1S, IL2RG, IQGAP2, IRS4, CREB3L1, GBP1, BANK1, RNA5SP111, RBM24, CNTN2 |
| Black | IGFBP6, TNNC1, DCN, CPNE7, ALDH1A3, SFRP4, TMEM255B, H19, PDE6C, PRELP, NGFR, PTGDS, UBE2QL1, RYR1, TMPRSS3, CCDC136, LONRF3, BRINP1, DM1-AS, FZD1, GAD2, CAPN12, MICAL2, APOD, JAG1, CRYM, SFRP5, AC009955.4, CYS1, ISL2, DYNLL1P1, FAM189A2, PLCE1, CLDN11, PDIA2, TUBB4A, EDNRB, PPFIA4, TRBV6-4, PLXNA2, BOK, LINC01432, SIX2, NOG, LHX3, HS6ST2 |

**Abbreviations:** SRG, stiffness-related gene.
