# Supplementary material for: Multi-Omics Investigations Revealed Underlying Molecular Mechanisms Associated With Tumor Stiffness and Identified Sunitinib as a Potential Therapy for Reducing Stiffness in Pituitary Adenomas
Source: Front Cell Dev Biol. 2022 Mar 15;10:820562. doi: 10.3389/fcell.2022.820562 (PMC8965615; doi:10.3389/fcell.2022.820562)
Supplement: Supplementary file 5 [file Table3.DOCX]

**Supplementary Table 3.** DMRs between soft and stiff PA samples.

| chr | start | end | length | nCG | meanMethy(Soft PA) | meanMethy(Stiff PA) | diff.Methy | areaStat |
| --- | --- | --- | --- | --- | --- | --- | --- | --- |
| chr9 | 38064001 | 38067001 | 3001 | 4 | 0.656834575 | 0.469302933 | 0.187531642 | 11.45248667 |
| chr8 | 26712001 | 26720001 | 8001 | 5 | 0.76125937 | 0.573998534 | 0.187260837 | 13.01267686 |
| chr17 | 27942001 | 27945001 | 3001 | 4 | 0.594942413 | 0.412194358 | 0.182748054 | 8.898925724 |
| chr8 | 37656001 | 37660001 | 4001 | 5 | 0.754358627 | 0.59432867 | 0.160029957 | 13.16236473 |
| chr5 | 134374001 | 134377001 | 3001 | 4 | 0.3358685 | 0.1760192 | 0.1598492 | 10.44136 |
| chr17 | 79376001 | 79380001 | 4001 | 5 | 0.850605675 | 0.691614339 | 0.158991335 | 13.63684276 |
| chr14 | 22979001 | 22984001 | 5001 | 4 | 0.796276488 | 0.649381573 | 0.146894915 | 9.067782826 |
| chr1 | 1365001 | 1369001 | 4001 | 5 | 0.752976789 | 0.606130706 | 0.146846083 | 11.79695255 |
| chr10 | 124902001 | 124905001 | 3001 | 4 | 0.267421898 | 0.120733252 | 0.146688646 | 9.182450394 |
| chr11 | 846001 | 849001 | 3001 | 4 | 0.473361973 | 0.32889756 | 0.144464413 | 53.34249764 |
| chr15 | 37386001 | 37390001 | 4001 | 5 | 0.223184094 | 0.081803096 | 0.141380999 | 11.27068055 |
| chr11 | 113344001 | 113349001 | 5001 | 6 | 0.561728342 | 0.420652172 | 0.14107617 | 14.13859248 |
| chr1 | 244263001 | 244270001 | 7001 | 4 | 0.878561061 | 0.744847738 | 0.133713323 | 9.914921611 |
| chr9 | 839001 | 843001 | 4001 | 5 | 0.578727984 | 0.44646601 | 0.132261974 | 29.11284554 |
| chr5 | 1469001 | 1473001 | 4001 | 4 | 0.660940411 | 0.539732396 | 0.121208014 | 37.99049971 |
| chr2 | 119913001 | 119916001 | 3001 | 4 | 0.336015819 | 0.215715673 | 0.120300145 | 10.95383966 |
| chr11 | 1942001 | 1947001 | 5001 | 5 | 0.761243186 | 0.641287798 | 0.119955388 | 16.1100017 |
| chr6 | 10718001 | 10724001 | 6001 | 4 | 0.703286414 | 0.584381975 | 0.118904439 | 18.55532805 |
| chr11 | 1223001 | 1230001 | 7001 | 7 | 0.620497246 | 0.508050241 | 0.112447005 | 99.7534627 |
| chr11 | 6339001 | 6342001 | 3001 | 4 | 0.7321666 | 0.6204853 | 0.1116812 | 9.046383 |
| chr12 | 10864001 | 10870001 | 6001 | 4 | 0.797429738 | 0.692598289 | 0.104831449 | 18.79183071 |
| chr19 | 38699001 | 38705001 | 6001 | 4 | 0.672788194 | 0.568111371 | 0.104676823 | 5.918669557 |
| chr7 | 1107001 | 1111001 | 4001 | 4 | 0.844806594 | 0.742758221 | 0.102048373 | 31.95739764 |
| chr9 | 134150001 | 134157001 | 7001 | 6 | 0.719315276 | 0.618433446 | 0.100881829 | 14.21514655 |
| chr1 | 3209001 | 3217001 | 8001 | 4 | 0.638562951 | 0.53778843 | 0.100774521 | 37.01194394 |
| chr2 | 23852001 | 23861001 | 9001 | 4 | 0.569680526 | 0.669869991 | -0.100189465 | -39.77900694 |
| chr11 | 61084001 | 61094001 | 10001 | 5 | 0.754299039 | 0.856607949 | -0.10230891 | -11.57451594 |
| chr10 | 426001 | 431001 | 5001 | 4 | 0.350051554 | 0.45616724 | -0.106115686 | -35.58139744 |
| chr6 | 166825001 | 166829001 | 4001 | 4 | 0.796598959 | 0.902790994 | -0.106192035 | -8.675072706 |
| chr4 | 745001 | 753001 | 8001 | 8 | 0.372626525 | 0.480808125 | -0.1081816 | -126.9918778 |
| chr2 | 487001 | 495001 | 8001 | 6 | 0.510879153 | 0.62156622 | -0.110687066 | -33.53765902 |
| chr2 | 1913001 | 1928001 | 15001 | 8 | 0.510623763 | 0.62257866 | -0.111954897 | -137.26721 |
| chr16 | 4815001 | 4819001 | 4001 | 4 | 0.467944111 | 0.58250426 | -0.114560148 | -19.75749839 |
| chr11 | 701001 | 713001 | 12001 | 10 | 0.583615594 | 0.705743422 | -0.122127828 | -186.4884231 |
| chrX | 197001 | 205001 | 8001 | 4 | 0.504244803 | 0.639872083 | -0.13562728 | -66.65662617 |
| chr11 | 520001 | 533001 | 13001 | 7 | 0.433193995 | 0.588319368 | -0.155125373 | -36.9803941 |
| chr8 | 1379001 | 1385001 | 6001 | 6 | 0.590903999 | 0.756140379 | -0.16523638 | -30.69212331 |
| chr17 | 17719001 | 17722001 | 3001 | 4 | 0.445888391 | 0.664498271 | -0.21860988 | -10.0980485 |

**Abbreviations:** DMR, differentially methylated region; PA, pituitary adenoma; chr, chromosome; nCG, number of CpG sites.
