# Supplementary material for: Multi-Omics Investigations Revealed Underlying Molecular Mechanisms Associated With Tumor Stiffness and Identified Sunitinib as a Potential Therapy for Reducing Stiffness in Pituitary Adenomas
Source: Front Cell Dev Biol. 2022 Mar 15;10:820562. doi: 10.3389/fcell.2022.820562 (PMC8965615; doi:10.3389/fcell.2022.820562)
Supplement: Supplementary file 6 [file Table4.DOCX]

**Supplementary Table 4.** Identification of potential drugs targeting the stiff PAs by CMap analysis.

| MoA | Perturbagen Count | Perturbagen Id | Name |
| --- | --- | --- | --- |
| VEGFR inhibitor | 14 | BRD-K29905972, BRD-K28428262, BRD-K86930074, BRD-K85402309, BRD-K68488863, BRD-K99749624, BRD-K95655893, BRD-K99616396, BRD-K74514084, BRD-K97764662, BRD-K49810818, BRD-M64432851, BRD-K53414658, BRD-K41337261 | axitinib, brivanib, cediranib, dovitinib, ENMD-2076, linifanib, MAZ-51, motesanib, pazopanib, PD-173074, sorafenib, sunitinib, tivozanib, ZM-306416 |
| Carbonic anhydrase inhibitor | 7 | BRD-K21450440, BRD-K74913225, BRD-A26384407, BRD-K71499074, BRD-K30649484, BRD-K13356952, BRD-K29653726 | benzthiazide, brinzolamide, chlortalidone, diclofenamide, mafenide, methazolamide, topiramate |
| PDGFR receptor inhibitor | 7 | BRD-K29905972, BRD-K85402309, BRD-K99749624, BRD-K99616396, BRD-K74514084, BRD-K49810818, BRD-M64432851 | axitinib, dovitinib, linifanib, motesanib, pazopanib, sorafenib, sunitinib |
| KIT inhibitor | 5 | BRD-K86930074, BRD-K99616396, BRD-K74514084, BRD-K49810818, BRD-M64432851 | cediranib, motesanib, pazopanib, sorafenib, sunitinib |
| FLT3 inhibitor | 4 | BRD-K85402309, BRD-K68488863, BRD-K49810818, BRD-M64432851 | dovitinib, ENMD-2076, sorafenib, sunitinib |
| FGFR inhibitor | 3 | BRD-K28428262, BRD-K85402309, BRD-K97764662 | brivanib, dovitinib, PD-173074 |
| Dopamine receptor antagonist | 2 | BRD-K37289225, BRD-K89732114 | clozapine, trifluoperazine |
| RET tyrosine kinase inhibitor | 2 | BRD-K49810818, BRD-M64432851 | sorafenib, sunitinib |
| Sodium/potassium/chloride transporter inhibitor | 2 | BRD-A80017228, BRD-K36862742 | bendroflumethiazide, hydroflumethiazide |
| ABL inhibitor | 1 | BRD-K41337261 | ZM-306416 |
| Acetylcholinesterase inhibitor | 1 | BRD-K81209159 | herniarin |
| Aurora kinase inhibitor | 1 | BRD-K68488863 | ENMD-2076 |
| Beta secretase inhibitor | 1 | BRD-K03600606 | catechin |
| Bile acid | 1 | BRD-K43164539 | cholic-acid |
| CALY activator | 1 | BRD-K79353516 | indolophenanthridine |
| carbonic anhydrase inhibitor | 1 | BRD-K14643723 | 4-(2-Amino-ethyl)-benzenesulfonamide |
| Cyclooxygenase inhibitor | 1 | BRD-K69690935 | curcumin |
| EGFR inhibitor | 1 | BRD-K85402309 | dovitinib |
| Estrogen receptor antagonist | 1 | BRD-K63828191 | raloxifene |
| Fatty acid synthase inhibitor | 1 | BRD-K03600606 | catechin |
| Glutamate receptor antagonist | 1 | BRD-K29653726 | topiramate |
| Histamine receptor antagonist | 1 | BRD-K67637637 | olopatadine |
| Histone acetyltransferase inhibitor | 1 | BRD-K69690935 | curcumin |
| Kainate receptor antagonist | 1 | BRD-K29653726 | topiramate |
| LDL antioxidant | 1 | BRD-K03600606 | catechin |
| Lipoxygenase inhibitor | 1 | BRD-K69690935 | curcumin |
| NFkB pathway inhibitor | 1 | BRD-K69690935 | curcumin |
| PLK inhibitor | 1 | BRD-K70511574 | sunitinib |
| RAF inhibitor | 1 | BRD-K49810818 | sorafenib |
| Selective estrogen receptor modulator (SERM) | 1 | BRD-K63828191 | raloxifene |
| Serotonin receptor antagonist | 1 | BRD-K37289225 | clozapine |
| Sodium channel blocker | 1 | BRD-K48300629 | zonisamide |
| SRC inhibitor | 1 | BRD-K41337261 | ZM-306416 |
| T-type calcium channel blocker | 1 | BRD-K48300629 | zonisamide |
| Vitamin K antagonist | 1 | BRD-K23913458 | coumarin |

**Abbreviations:** PA, pituitary adenoma; CMap, Connectivity Map; MoA, mode of action.
